# Astrocytes respond to a neurotoxic Aβ fragment with state-dependent Ca^2+^ alteration and multiphasic transmitter release

**DOI:** 10.1186/s40478-021-01146-1

**Published:** 2021-03-16

**Authors:** Cuong Pham, Karine Hérault, Martin Oheim, Steeve Maldera, Vincent Vialou, Bruno Cauli, Dongdong Li

**Affiliations:** 1 Institute of Biology Paris Seine, Neuroscience Paris Seine, CNRS UMR8246, INSERM U1130, Sorbonne Université, 75005 Paris, France; 2 INSERM/UdA U1107 Neuro-Dol, Université Clermont Auvergne, 63100 Clermont-Ferrand, France; 3 Saints-Pères Paris Institute for the Neurosciences (SPPIN), CNRS UMR8003, Université de Paris, 75006 Paris, France

**Keywords:** ATP, Glutamate, Hemichannel, Lysosome, Alzheimer’s disease

## Abstract

Excessive amounts of amyloid β (Aβ) peptide have been suggested to dysregulate synaptic transmission in Alzheimer’s disease (AD). As a major type of glial cell in the mammalian brain, astrocytes regulate neuronal function and undergo activity alterations upon Aβ exposure. Yet the mechanistic steps underlying astrocytic responses to Aβ peptide remain to be elucidated. Here by fluorescence imaging of signaling pathways, we dissected astrocytic responses to Aβ25–35 peptide, a neurotoxic Aβ fragment present in AD patients. In native health astrocytes, Aβ25–35 evoked Ca^2+^ elevations via purinergic receptors, being also dependent on the opening of connexin (CX) hemichannels. Aβ25–35, however, induced a Ca^2+^ diminution in Aβ-preconditioned astrocytes as a result of the potentiation of the plasma membrane Ca^2+^ ATPase (PMCA). The PMCA and CX protein expression was observed with immunostaining in the brain tissue of hAPPJ20 AD mouse model. We also observed both Ca^2+^-independent and Ca^2+^-dependent glutamate release upon astrocytic Aβ exposure, with the former mediated by CX hemichannel and the latter by both anion channels and lysosome exocytosis. Our results suggest that Aβ peptide causes state-dependent responses in astrocytes, in association with a multiphasic release of signaling molecules. This study therefore helps to understand astrocyte engagement in AD-related amyloidopathy.

**Supplementary Information:**

The online version contains supplementary material available at 10.1186/s40478-021-01146-1.

## Introduction

Toxic Aβ peptides are implicated in the development of cognitive deficits of AD [10]. In the mammalian brain, information processing is sustained by dynamic interactions between neurons and glial cells [13]. Emerging evidence suggests that Aβ dysregulates neuron-glia communication thereby impairing synaptic transmission [33]. In the meanwhile, therapeutics targeting neuronal dysfunctions yield only limited effects [32], urging the need to examine Aβ-caused pathological adaptations in glial signaling.

Astrocytes are the major glial cell-type in the mammalian brain [34]. Albeit electrically non-excitable, their activity is encoded by intracellular Ca^2+^ signaling [43], which in turn modulates neuron activity, via for instance regulating ambient transmitter and ion recycling [19, 91], the delivery of energy fuels [76], the peri-synaptic structural remodeling [69] as well as the release of transmitter substances [5]. Astrocyte Ca^2+^ activity has been shown to be upregulated by Aβ peptides [2, 11, 85], and near amyloid plaques in AD mouse models [17, 47]. Aβ-caused Ca^2+^ hyperactivity was found to compromise neuronal survival [1]. Accordingly, inhibiting the Ca^2+^-dependent protein phosphatase calcineurin in astrocytes ameliorates synaptic function in AD mouse model [27]. In addition, the cognitive deficit during AD progression has been attributed to synapse excitotoxicity, a process involving aberrated astrocytic handling of neurotransmitter recycling and signaling molecule release [63, 64, 90, 98]. While astrocytes are being recognized to participate in AD amyloidopathy, the dynamically weaved signaling cascades remain to be delineated.

Here, we imaged astrocytic signaling cascades in response to Aβ25–35, a neurotoxic Aβ fragment found in AD patients [4, 46, 57]. We observed that Aβ25–35 upregulated Ca^2+^ signals in primary astrocytes derived from mouse cortex, which involved the activation of metabotropic P2Y receptor and the opening of CX hemichannel. In contrast, Aβ25–35 caused a Ca^2+^ diminution in Aβ-preconditioned astrocytes that involved the potentiated Ca^2+^ extrusion via PMCA and the activation of cAMP signal. We further observed both Ca^2+^-independent and -dependent glutamate release in astrocytes upon Aβ25–35 exposure, which relied respectively on CX hemichannel, anion channels and lysosome exocytosis. These results show a state-dependent adaptation in astrocyte responses to neurotoxic Aβ peptide, and suggest molecular targets to control astrocyte functions in AD amyloidopathy.

## Materials and methods

### Animals and preparation of primary cortical astrocytes

Our laboratory follows the European Union and institutional guidelines for the care and use of experimental animals (Council directive 86/609EEC). The care of experimental animals was also in conformity with the French National Charter on the ethics of animal experimentation. Primary astrocytes were cultured from the neocortex of P0-1 NMRI mice of either sex adapted from the published protocol [52, 56]. Astrocytes were plated in Petri dishes for 1 week prior to being transferred to cover slips (#1, BK-7, 25-mm, Menzel-Gläser) coated with poly-ornithine (Sigma). Cells were kept at 37 °C in a humidified 5% CO_2_ atmosphere in Dulbecco's Modified Eagle Medium (DMEM, Invitrogen) supplemented with 5% fetal bovine serum (FBS, HyClone), penicillin (5 U/ml, Sigma), and streptomycin (5 μg/ml, Sigma). Recordings were made during the following week at room temperature (RT, 22–23 °C) in the standard extracellular saline containing (in mM): 140 NaCl, 5.5 KCl, 1.8 CaCl_2_, 1 MgCl_2_, 20 glucose, 10 HEPES (pH 7.3, adjusted with NaOH). The hAPPJ20 AD mouse model was obtained from The Jackson Lab (No: 34836-JAX), expressing mutated human amyloid precursor protein (hAPP) monogene comprising the Swedish (K670N/M671L) and the Indiana (V717F) mutations [44, 60]. The overexpression of the mutated hAPP was controlled under the human platelet-derived growth factor beta polypeptide promoter. This AD mouse model displays diffusive Aβ peptides at age ~ 5–7 months and plaques by age ~ 8–10 months [44, 60]. Breeding was made between hemizygote males and C57BL/6 females to obtain hAPPJ20 mice (~ 7 month old) for slice immunohistochemistry as stated below.

### Fluorophores and drugs

The chemical Ca^2+^-indicator dyes Oregon Green BAPTA-1 AM (OGB-1 AM) or Xrhod-1 AM (Invitrogen) were loaded into astrocytes by incubating them in dye-containing extracellular solutions (2 µM, 40 min for OGB-1; 200 nM, 10 min for Xrhod-1, respectively). To label lysosomes, astrocytes were incubated in 6.7 µM FM4-64 (Invitrogen) for 30 min. To fluorescently label ATP accumulation compartments in live cells, astrocytes were incubated in 50 µM MANT-ATP (Invitrogen) for 1 h. Prior to live cell imaging, cells were thoroughly washed during at least 30 min, and they were continuously perfused by dye-free solution (~ 0.5 ml/min) during imaging. BAPTA AM (Invitrogen; 100 µM for 50 min) was used to chelate astrocyte intracellular Ca^2+^. Plasmids encoding fluorescent sensors were obtained from Addgene unless otherwise indicated. Lipofectamine 2000 (Invitrogen) was used for transfecting cDNA plasmids into astrocytes following the standard protocol provided by the supplier. Cells were used ~ 24 h after transfection. Suramin, MPEP, thapsigargin and Gap26 were purchased from Tocris, 2-APB from Ascent Scientific, forskolin from Abcam, Aβ25–35 from Bachem, and all other compounds from Sigma-Aldrich. Dual-channel local perfusion system was used to switch smoothly between control solution and specific pharmacological manipulations, and controlled by electric valves operated by a TTL trigger box commanded by MetaMorph (Molecular Devices). Immunostaining for mouse brain slices was performed following the standard protocol as previously described [70]. As for immunostaining of astrocyte primary cultures, cells were fixed with 1% paraformaldehyde (PFA, Sigma-Aldrich) for 10 min at RT, then washed three times with phosphate buffered saline (PBS, 5 min, at RT). After permeabilization and blockage of unspecific sites with PBS, 0.3% Triton X-100 and 2% bovine serum albumin (PBS-BT, 1 h at RT), astrocytes were probed with respective primary antibodies in the same solution overnight at 4 °C. After being washed with PBS three times at RT, cells were incubated with secondary antibodies in PBS-BT (2 h, RT). After three times of final washing (PBS, 5 min, RT) and rinsed afterwards with triple distilled water, cells were mounted with Mowiol (Millipore, Darmstadt, Germany) onto microscope slides. Combinations of the primary and secondary antibodies used for fluorescence immunostaining are listed in Additional file 1: Table S1.

### Fluorescence imaging and analysis

Total internal reflection fluorescence (TIRF) imaging was performed on a custom-made inverted microscope via the through-the-objective configuration (PlanApo TIRF × 60/NA1.45 oil objective, Olympus) [61]. The 488- and 568-nm excitation wavelengths were isolated from the beam of an Ar^+^/Kr^+^ multi-line laser (CVI Melles Griot) with an acousto-optical tunable filter (AA.Opto). Laser beam was directed onto the glass/water interface at a super-critical angle, thereby enabling the total reflection of the excitation beam and the generation of evanescent field on the side of astrocyte substrate. The penetration depth (1/e^2^-intensity decay) of the evanescent field was estimated of the order of 200 nm [61], thereby allowing ultrathin optical sectioning in astrocyte subplasmalemmel region for dynamic signal recording. Emission fluorescence was further magnified (× 2) and acquired by an electron multiplying charge-coupled device (EMCCD, QuantEM 512, Princeton Instruments), and the effective pixel size in fluorescent images was 133 nm. The imaging hardwares were all controlled by MetaMorph software (Molecular Devices). For TIRF imaging, each field of view in general contains the footprint of a single astrocyte. In our recording, two to three separate astrocyte culture preparations were used and about three independent coverslips of each preparation for TIRF imaging per condition. Results were derived from signals of all recorded cells.

Background was estimated from the autofluorescence signal in non-labeled cells of the same preparation, and then subtracted from the fluorescent images. The contour of the footprint of single astrocytes was delineated with the ImageJ plugin Cell Outliner or with MetaMorph segmentation tool, from which the mean fluorescence was measured over time to generate the time courses of specific signals. The FRET ratio of the cAMP sensor GFP(nd)-EPAC1(dDEP)-mCherry and the donor/acceptor bleed-through control was obtained as previously reported [71]. During TIRF imaging, the 488-nm laser line was used to excite the cAMP sensor, while both GFP and mCherry fluorescence were simultaneously collected and projected by a custom image splitter side-by-side onto a single EMCCD camera. Details of the optical filter set are listed in Additional file 1: Table S2. Corrected by the amount of acceptor direct excitation and donor bleed-through [71], the FRET signal was calculated from the GFP/mCherry ratio and normalized to the pre-stimulation basal level as fractional changes.

### Statistics

All data are expressed as mean ± standard deviation (SD), and the t-test was used for assessing the significance. Comparison of non-normally distributed data was also validated using their median ± absolute deviation and the non-parametric tests (Kolmogorov–Smirnov or Mann–Whitney U-test). All statistical operations were performed with Matlab (The MathWorks), with n.s., denoting non significant, *p < 0.05, and **p < 0.01.

## Results

### Astrocytic Ca^2+^ elevation induced by neurotoxic Aβ25–35

**Fig. 1:**
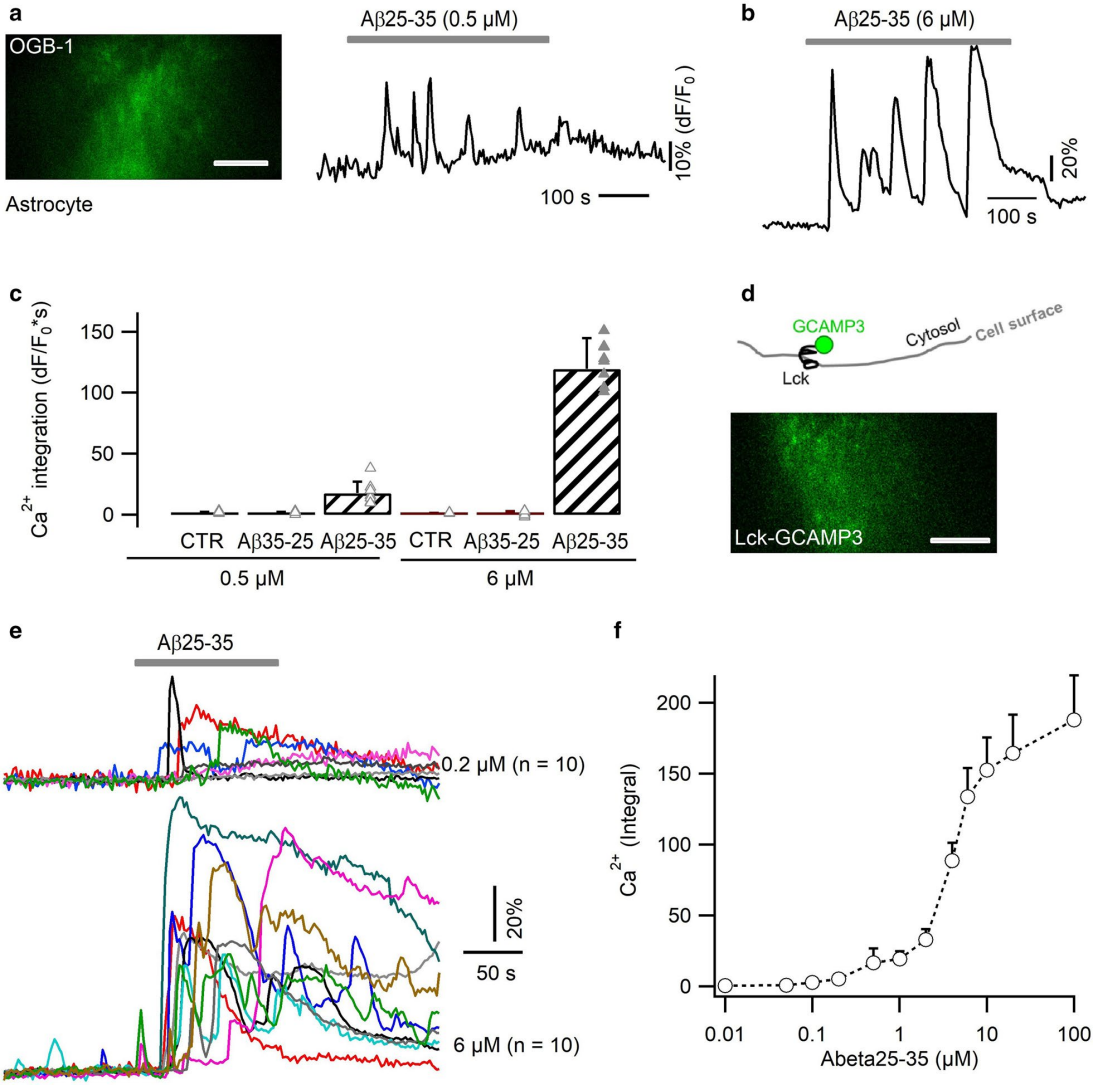
Neurotoxic Aβ25–35 peptide triggered irregular Ca^2+^ rises in primary astrocytes. **a**, **b** Aβ-triggered Ca^2+^ transients in primary cultures of mouse cortical astrocytes, imaged with the chemical Ca^2+^ indicator OGB-1 AM. Subplasmalemmal Ca^2+^-dependent fluorescence changes were selectively imaged by TIRFM. **c** Dose–response of the Aβ25–35 effect. The strength of Ca^2+^ signals was evaluated by their temporal integral over the same recording period (n = 8–13 astrocytes per condition). **d** Lck-GCaMP3 was expressed on the inner side of the astrocytic plasma membrane. Below, representative TIRFM image. **e** Astrocytic Ca^2+^ signals evoked by 0.2 µM and 6 µM Aβ25–35, respectively. Each trace denotes the response from a single cell. **f** Dose-responses curve of astrocyte Ca^2+^ response to Aβ peptide (n = 7–12 per condition). Scale bars, 10 µm

To study the acute response of astrocytes to Aβ, we used TIRF microscopy (TIRFM) to image near-membrane Ca^2+^ transients in primary astrocytes cultured from mouse cortex. With the cytosolic Ca^2+^ indicator OGB-1 AM, we observed an oscillatory Ca^2+^ increase upon the local application of submicromolar Aβ25–35 (0.5 µM, temporal integral = 17.6 ± 9.5 dF/F_0_*s; Fig. 1a). Higher doses of Aβ evoked stronger (6 µM, integral = 119.5 ± 25.4 dF/F_0_*s) and longer-lasting Ca^2+^ signals following a temporal delay (43.5 ± 21.6 s; Fig. 1b, c). This signal was absent in response to extracellular control solutions, either without Aβ peptide or containing the sequence-reversed peptide Aβ35–25 (6 µM, Additional file 1: Fig. S1a). To further confine Ca^2+^ detection in subplasmalemmal region, we also used the plasma membrane-targeted Ca^2+^ sensor Lck-GCaMP3 [80] (Fig. 1d, e). As before, Ca^2+^ elevations could be detected in response to submicromolar Aβ25–35 and gradually reached to a plateau level with increased doses (Fig. 1f). The similarity in Ca^2+^ profiles detected with the bulk indicator OGB-1 and the subplasmalemmal sensor Lck-GCaMP3, suggests that the neurotoxic Aβ25–35 causes Ca^2+^ elevations throughout astrocyte cytosol.

### Purinergic activation contributes to Aβ-evoked astrocytic Ca^2+^ rise

**Fig. 2:**
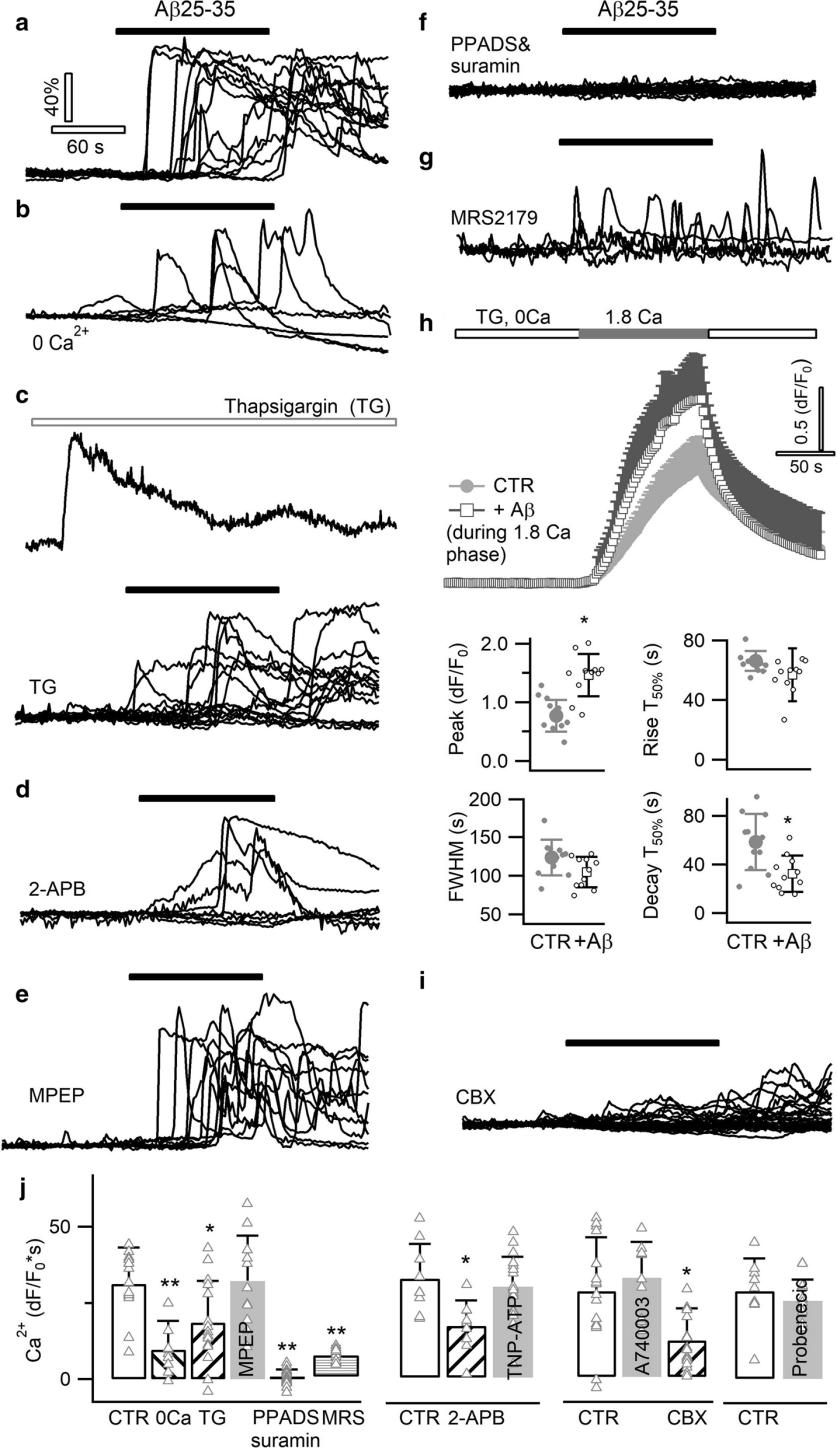
A purinergic pathway underlies Aβ-evoked Ca^2+^ signal. Representative responses evoked by Aβ25–35 in astrocytes loaded with the chemical Ca^2+^ indicator OGB-1 AM, in control condition (**a**), in Ca^2+^-free extracellular solution (**b**), following thapsigargin (TG, 0.5 µM) depletion of ER Ca^2+^ store (**c**, top trace reflecting the Ca^2+^ leak signal upon TG application), in the presence of the IP3 receptor antagonist 2-APB (**d**, 200 µM), and of the mGluR5 antagonist MPEP (**e**, 50 µM). Each trace represents the response of a single astrocyte. **f** Aβ-evoked Ca^2+^ responses were fully abolished by blocking purinergic P2 receptors with the combination of wide-spectrum antagonists PPADS (100 µM) and suramin (50 µM). **g** The P2Y1 antagonist MRS2179 (5 µM) attenuated Aβ-induced Ca^2+^ responses. **h** Aβ25–35 enhanced Ca^2+^ influx via store-operated channels (SOCs). SOCs were activated by fully depleting the ER store with TG in Ca^2+^-free solution. SOC-mediated Ca^2+^ influx was induced by re-supplying Ca^2+^ in the extracellular solution. Ca^2+^ influx was significantly increased in the presence of Aβ25–35 (n = 11 cells per condition). **i** Effect of blocking connexin hemichannels with CBX (50 µM). **j** Aβ-evoked astrocyte Ca^2+^ responses in different conditions. Ca^2+^ signal strength was derived from the temporal integral of individual normalized traces (dF/F_0_*s). Wide-spectrum P2X receptor antagonist TNP-ATP, P2X7 antagonist A740003 and pannexin blocker probenecid were applied at 10 µM, 20 µM and 500 µM, respectively. Control experiments were performed for a defined set of experiments as shown (n = 9–20 cells per condition)

Aβ peptides have been suggested to induce Ca^2+^ influx [1, 18], while other studies showed the contribution of intracellular Ca^2+^ release from the endoplasmic reticulum (ER) store [3, 31, 85]. We therefore examined in primary astrocytes the mechanism for Aβ25–35-evoked Ca^2+^ rise. Compared to control condition (temporal integral dF/F_0_*s = 31.2 ± 11.9; Fig. 2a, j), removing Ca^2+^ from extracellular solution diminished the Aβ25–35-evoked Ca^2+^ signal (integral = 9.7 ± 10.1, *p* < 0.01; Fig. 2b, j). We then kept the extracellular Ca^2+^ at normal level while pre-depleting the internal ER Ca^2+^ store. To this end, the ER-resident Ca^2+^ ATPase was inhibited by thapsigargin (TG, 0.5 µM), which caused a prominent discharge of Ca^2+^ from ER store (Fig. 2c, top). This treatment significantly decreased the subsequent responses to Aβ25–35 (integral = 18.5 ± 13.7%, *p* < 0.05; Fig. 2c, bottom; j). Since Ca^2+^ release from ER store is mediated by the inositol 1,4,5-trisphosphate (IP3) receptor, we examined the effect of its blocker 2-Aminoethoxydiphenyl borate (2-APB, 200 µM) and we also observed an inhibition impact (integral = 17.8 ± 9.6, *p* < 0.05; Fig. 2d, j). Thus, both Ca^2+^ influx and release from the ER store contribute to the Aβ-evoked astrocytic Ca^2+^ signal.

Ca^2+^ release from the internal store is recruited by the activation of metabotropic receptors. Astrocytes express a variety of receptors, among which metabotropic glutamate receptor 5 (mGluR5) and purinergic P2 receptors respond with Ca^2+^ rises to glutamate and ATP, respectively [6]. While antagonizing mGluR5 with the group I mGluR antagonist MPEP showed no effect (50 µM, *p* = 0.6; Fig. 2e, j), Aβ-evoked Ca^2+^ signals were fully abolished by the combination of P2 receptor antagonists PPADS (100 µM) and suramin (50 µM, *p* < 0.01; Fig. 2f, j). This indicates that Aβ-induced Ca^2+^ increase requires the activation of astrocytic P2 receptors. Although both ionotropic P2X and metabotropic P2Y receptors were suggested to regulate astrocytic Ca^2+^ signal, we observed that the wide-spectrum P2X antagonist TNP-ATP [41] failed to inhibit Aβ-triggered Ca^2+^ rise (10 µM, *p* = 0.53, Fig. 2j). In contrast, the Aβ response was inhibited by antagonizing the P2Y1 receptor (5 µM MRS2179, integral = 17.5 ± 7.8, *p* < 0.01; Fig. 2g, j), in line with its contribution to astrocyte Ca^2+^ hyperactivities in AD mouse model [17].

Metabotropic receptor activation triggers Ca^2+^ release from internal ER store, which then activates store-operated channels (SOCs) to induce Ca^2+^ influx [83]. Lipophilic molecules including Aβ peptide were suggested to facilitate Ca^2+^ influx through astrocytic SOCs [74, 88]. To image SOC-mediated Ca^2+^ upon, we used a standard protocol to image SOC-mediated Ca^2+^ influx [83]. ER store was first depleted by thapsigargin in Ca^2+^-free solution, and then Ca^2+^ added back to generate SOC-mediated Ca^2+^ influx (Fig. 2h). Presence of Aβ25–35, indeed, facilitated SOC Ca^2+^ influx (peak amplitude, dF/F_0_ = 1.46 ± 0.36 vs. CTR 0.77 ± 0.27, *p* < 0.05; Fig. 2h), suggesting its contribution to the Ca^2+^ oscillations following P2Y1 receptor activation. These results corroborate the dual dependence of Aβ-evoked Ca^2+^ signal on both the internal store and Ca^2+^ influx (Fig. 2b, c, j).

One possible mechanism underlying P2Y1 activation by Aβ might be that it activated ATP-releasing pathways in astrocytes. In spinal cord and hippocampal astrocytes, ATP release was suggested to be mediated by the pore-forming P2X7 receptor [95], although its expression in astrocytes of specific regions was called into reconsideration [59]. We observed that P2X7 antagonist A740003 (20 µM) [35] failed to affect Aβ-triggered Ca^2+^ signal, echoing the absence of an effect of the wide-spectrum P2X blocker TNP-ATP (Fig. 2j). Alternatively, astrocytes express connexin (CX) hemichannels that mediate ATP release in physiological and pathological conditions [28, 98]. Immunostaining of CX43 protein was observed on the surface of cortical astrocytes in culture and in the cortex of hAPPJ20 AD mouse model [44, 60] (Additional file 1: Fig. S2). We found that blocking CX hemichannels with carbenoxlone (CBX, 50 µM) reduced Aβ-elicited Ca^2+^ oscillation (integral = 13.2 ± 15.5 vs. CTR 37.5 ± 24.9, *p* < 0.05; Fig. 2i, j). Although astrocyte ATP was also shown to be released by pannexin hemichannel [36], its blocker probenecid (500 µM) failed to alter the Aβ25–35 effect (Fig. 2j). Hence, Aβ-caused Ca^2+^ oscillaitons depend on the opening of CX hemichannels, by which ATP might be released to activate the P2Y1 purinergic receptor.

### Aβ diminishes Ca^2+^ level in preconditioned astrocytes

**Fig. 3:**
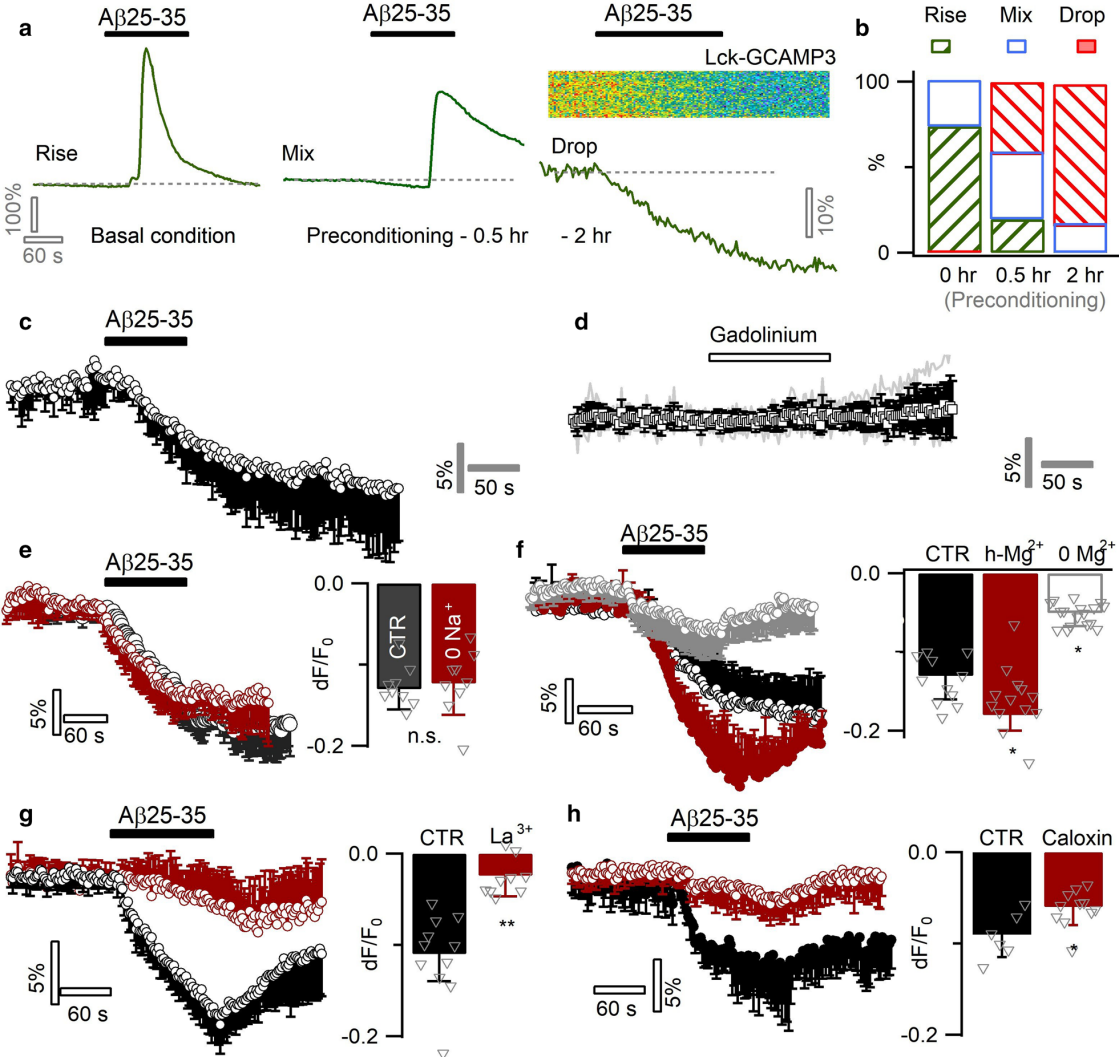
Aβ25–35 inhibits Ca^2+^ levels in preconditioned astrocytes by potentiating PMCA Ca^2+^ extrusion. **a** Effect of preconditioning on the acute responses of Aβ25–35 (6 µM) in cultured mouse cortical astrocytes. Left, Ca^2+^ rise was triggered in intact astrocytes (‘Rise’ type response). Middle, after a short term incubation (i.e., preconditioning) of astrocytes with submicromolar Aβ25–35 (0.5 h, 0.5 µM), acute application of 6 µM Aβ25–35 caused a basal line drop mixed with Ca^2+^ rise (‘Mix’ type response). Right, following a ~ 2 h preconditioning in 0.5 µM Aβ25–35, astrocytes exhibited only a drop in the intracellular Ca^2+^ level (‘Drop’ type response). **b** The percentage of three types of astrocytes that either displayed a Ca^2+^ rise (‘Rise’), an initial diminution followed by rise (‘mix’), or only a drop in the basal Ca^2+^ levels (Drop’; n = 19–7 cells per condition). **c** Average of Aβ-induced Ca^2+^ diminution in Aβ25–35-preconditioned (2 h) astrocytes (n = 11). **d** Blocking spontaneous Ca^2+^ influx by gadolinium (100 µM) failed to mimic Aβ-evoked Ca^2+^ diminution (n = 8). **e** Inactivating NCX by Na^+^-free solution showed no effect (n = 9 per condition). **f** Aβ-induced Ca^2+^ diminution affected by ambient Mg^2+^ concentration, implying the recruitment of an ATP-dependent pathway. Astrocytes were incubated in defined solutions 1 h prior to imaging (n = 11–17 per condition). **g**, **h** Inhibiting PMCA by La^3+^ (50 µM) or Caloxin 3A1 (500 µM) counterbalanced the Aβ-evoked astrocytic Ca^2+^ diminution (n = 6–11 per condition)

Astrocytes change status over chronic Aβ exposure [4, 64]. In AD patients and mouse models, astrocytes become reactive near Aβ plaques [55]. To examine potential response alterations in Aβ-conditioned astrocytes, we pre-incubated primary astrocyte cultures with submicromolar Aβ25–35 (0.5 µM) for different durations. Such preconditioning reduced the number of astrocytes displaying Ca^2+^ rises (‘Rise’ type response, Fig. 3a *left*) in response to the subsequent acute Aβ25–35 challenge, and over time started diminishing the basal Ca^2+^ level (‘Mix’ type resposne, Fig. 3a *middle*). After a 2-h preconditioning, most astrocytes responded to Aβ25–35 with a diminution in basal Ca^2+^ level (‘Drop’ type response, Fig. 3a *right*; Fig. 3b, versus control Additional file 1: Fig. S1b). Spontaneous Ca^2+^ rises that occurred in a subpopulation of pre-conditioned astrocytes (16/117 cells), were also inhibited by an acute application of Aβ25–35 (Additional file 1: Fig. S1c, d).

We then probed potential mechanisms underlying the inhibition effect of Aβ. Spontaneous Ca^2+^ entry via SOC and the transient receptor potential cation channel A1 (TRPA1) channel regulates basal Ca^2+^ level in astrocytes [81, 83]. The inhibition effect of Aβ25–35 might be due to the blockade of this spontaneous Ca^2+^ entry. Yet blocking SOC and TRPA1 channels by the wide-spectrum blocker gadolinium (Gd^3+^, 100 µM) [72, 81] failed to mimic the effect of Aβ25–35 (Fig. 3c, d). Alternatively, the Ca^2+^ diminution might be due to the potentiation of Ca^2+^ extrusion. We noted that chronic pre-conditioning of astrocytes with low Aβ25–35 gradually elevated intracellular Ca^2+^ level (Additional file 1: Fig. S1e). The overload of Ca^2+^ would likely facilitate its extrusion due to the increased efflux driving force. Potential extrusion pathways include Na^+^/Ca^2+^ exchanger (NCX) that utilizes the Na^+^ gradient to export intracellular Ca^2+^ and the plasma membrane Ca^2+^ ATPase (PMCA) driven by ATP hydrolysis [7, 26, 73]. Inhibiting NCX with Na^+^-free external solution showed no effect on Aβ-caused Ca^2+^ diminution (dF/F_0_ = -12.6 ± 6.7% vs. CTR dF/F_0_ = −12.9 ± 5.6%, *p* = 0.7; Fig. 3e). We then examined the role of PMCA ATPase. As ATP requires the binding to Mg^2+^ to become biologically active in the form Mg-ATP [30, 92], we sought to up- and down-regulate PMCA activity by bathing astrocytes, respectively, in high (20 mM) or zero concentration of extracellular Mg^2+^ (vs. CTR, 1 mM). This manipulation proportionally altered Aβ-induced Ca^2+^ decline (Fig. 3f). Further, the wide-spectrum PMCA blocker La^3+^ (50 µM) [7, 14, 82], largely antagonized the Ca^2+^ diminution induced by Aβ25–35 (dF/F_0_ = −2.4 ± 3.7% vs. CTR dF/F_0_ = −11.3 ± 4.9%, *p* < 0.01; Fig. 3g). A similar effect was observed with the PMCA-blocking peptide caloxin 3A1 [22, 65] (500 µM, dF/F_0_ = −5.9 ± 1.9% vs. CTR dF/F_0_ = −9.1 ± 2.3%, *p* < 0.05; Fig. 3h). As PMCA exports Ca^2+^ against its transmembrane gradient, we expected this process to be facilitated by the removal of extracellular Ca^2+^. Indeed, Ca^2+^-free external solution largely augmented the Aβ-potentiated Ca^2+^ efflux (Additional file 1: Fig. S1f). The expression of PMCA in astrocytes was observed by immunohistochemistry. Astrocytes characterized by GFAP immunostaining could be observed in the cortical region of a hAPPJ20 AD mouse model [44, 60] (Additional file 1: Fig. S3a). We observed PMCA immunostaining in GFAP-positive astrocytes in culture (Additional file 1: Fig. S3b). PMCA expression was also present in cortical astrocytes identified by a wide-spectrum marker S100β (Additional file 1: Fig. S3c), suggesting a general involvement of PMCA in astrocyte activity regulation. These results, together, suggest that Aβ25–35 potentiates PMCA-mediated Ca^2+^ extrusion in preconditioned astrocytes, thereby diminishing the intracellular Ca^2+^ level.

### Aβ activates PMCA Ca^2+^–H^+^ exchange via cAMP signal

**Fig. 4:**
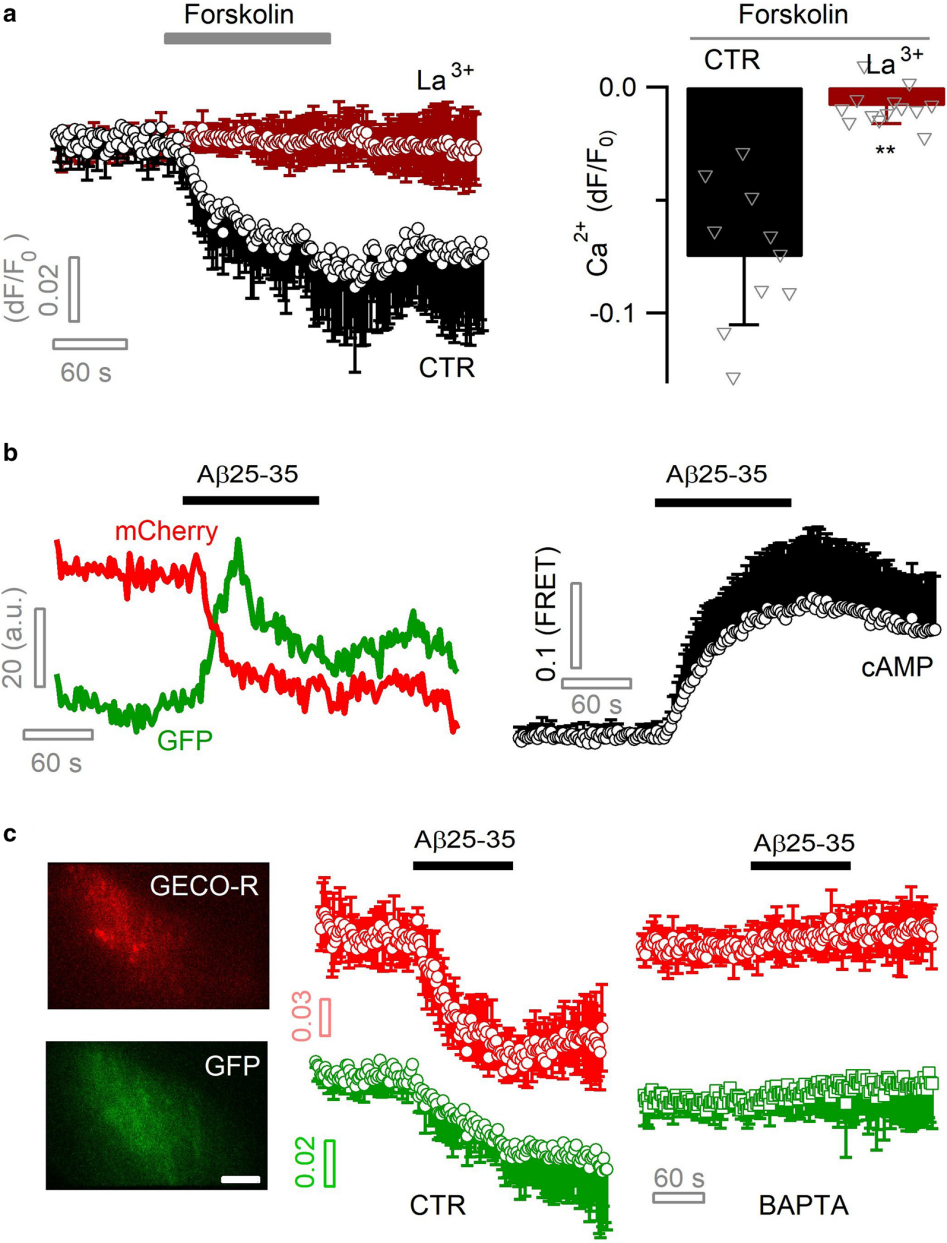
Aβ potentiates astrocytic PMCA via cAMP signaling. **a** Elevation of astrocytic cAMP level increased PMCA-mediated Ca^2+^ extrusion. cAMP was elevated by forskolin (100 µM) and sub-cellular Ca^2+^ level imaged with Lck-GCaMP3 and TIRFM. Ca^2+^ diminution was blocked by the PMCA blocker La^3+^ (50 µM; n = 9–13 cells per condition). **b** Intracellular cAMP level was monitored with the FRET sensor GFP^nd^-EPAC(dDEP)-mCherry and dual-color TIRFM. Left, representative dual-color recording of fluorescence change for EGFP and mCherry of the FRET sensor. Right, averaged astrocytic cAMP rise induced by Aβ25–35 (6 µM; n = 6). **c** PMCA-mediated astrocytic Ca^2+^ diminution was coupled with H^+^ influx. Dual-color TIRFM recorded concomitant cytosolic Ca^2+^ and pH diminution, by expressing the red genetically encoded Ca^2+^ sensor GECO-R and the pH-sensitive GFP protein in same astrocytes. Both effects were abolished by chelating astrocyte cytoplasmic Ca^2+^ with BAPTA AM (100 µM; n = 7–9 cells per condition). Scale bar, 5 µm

We next examined the signaling link between Aβ25–35 and PMCA potentiation. PMCA is activated upon intracellular rise of Ca^2+^ so as to prevent its overload [7, 62]. In preconditioned astrocytes, Aβ25–35 diminished basal Ca^2+^ level independent of its elevation, suggesting other signaling pathways than Ca^2+^ had been recruited. Another second messenger cAMP is also known to potentiate PMCA activity [16, 40]. Indeed, elevating cytosolic cAMP by forskolin (100 µM) induced a diminution in basal Ca^2+^ level (peak dF/F_0_ = −7.5 ± 3.1%) in primary cultured astrocytes, which was counteracted by the PMCA blocker La^3+^ (50 µM, dF/F_0_ = −0.82 ± 0.7%, *p* < 0.01; Fig. 4a). Forskolin also inhibited the spontaneous Ca^2+^ transients observed in a subpopulation of preconditioned astrocytes (Additional file 1: Fig. S1g), as seen with Aβ25–35 (Additional file 1: Fig. S1c, d). By a fluorescent FRET sensor GFP^nd^‐EPAC(dDEP)‐mCherry [49, 87], astrocytic cAMP increase was detected upon Aβ25–35 application (Fig. 4b). These results suggest that cAMP signal is involved in Aβ potentiation of PMCA Ca^2+^ extrusion.

PMCA couples Ca^2+^ export with H^+^ influx [7, 14]. In preconditioned astrocytes, we therefore performed dual-color imaging to simultaneously follow intracellular pH and Ca^2+^ dynamics during Aβ application. As the fluorescence of GFP protein can be quenched by H^+^ [45], we used it here as a pH sensor. Meanwhile, we co-expressed a red-fluorescent Ca^2+^ sensor protein GECO-R [102] to image cytoplasmic Ca^2+^. We observed a concomitant decrease in GFP fluorescence (dF/F_0_ = −4.0 ± 3.7%) upon Aβ25–35-induced Ca^2+^ diminution (dF/F_0_ = −7.1 ± 2.3%; Fig. 4c), indicating PMCA-mediated H^+^ influx accompanying the Ca^2+^ extrusion. Consistently, such phenomenon was abolished by chelating cytosolic Ca^2+^ with 100 µM BAPTA AM (GFP, dF/F_0_ = 1.1 ± 1.7%, *p* < 0.01; Fig. 4c). In preconditioned astrocytes that displayed mixed Ca^2+^ responses (i.e.,* an initial Ca*^2+^ *diminution followed by elevation*) to Aβ25–35, we observed stepwise H^+^ influx recorded by GFP quenching (Additional file 1: Fig. S1h) confirming the recruitment of PMCA by both Ca^2+^ phases. It is worth noting that H^+^ is a potent inhibitor of astrocytic CX hemichannels [77]. In preconditioned astrocytes, Aβ25–35 pontentiates PMCA causing Ca^2+^ diminution and H^+^ influx that would have inhibited CX hemichannels, an effect found to inhibit Ca^2+^ rises (Fig. 2i, j). Astrocytes hence display state-dependent Ca^2+^ responses to the neurotoxic Aβ25–35.

### Aβ25–35 evokes biphasic glutamate release from astrocytes

**Fig. 5:**
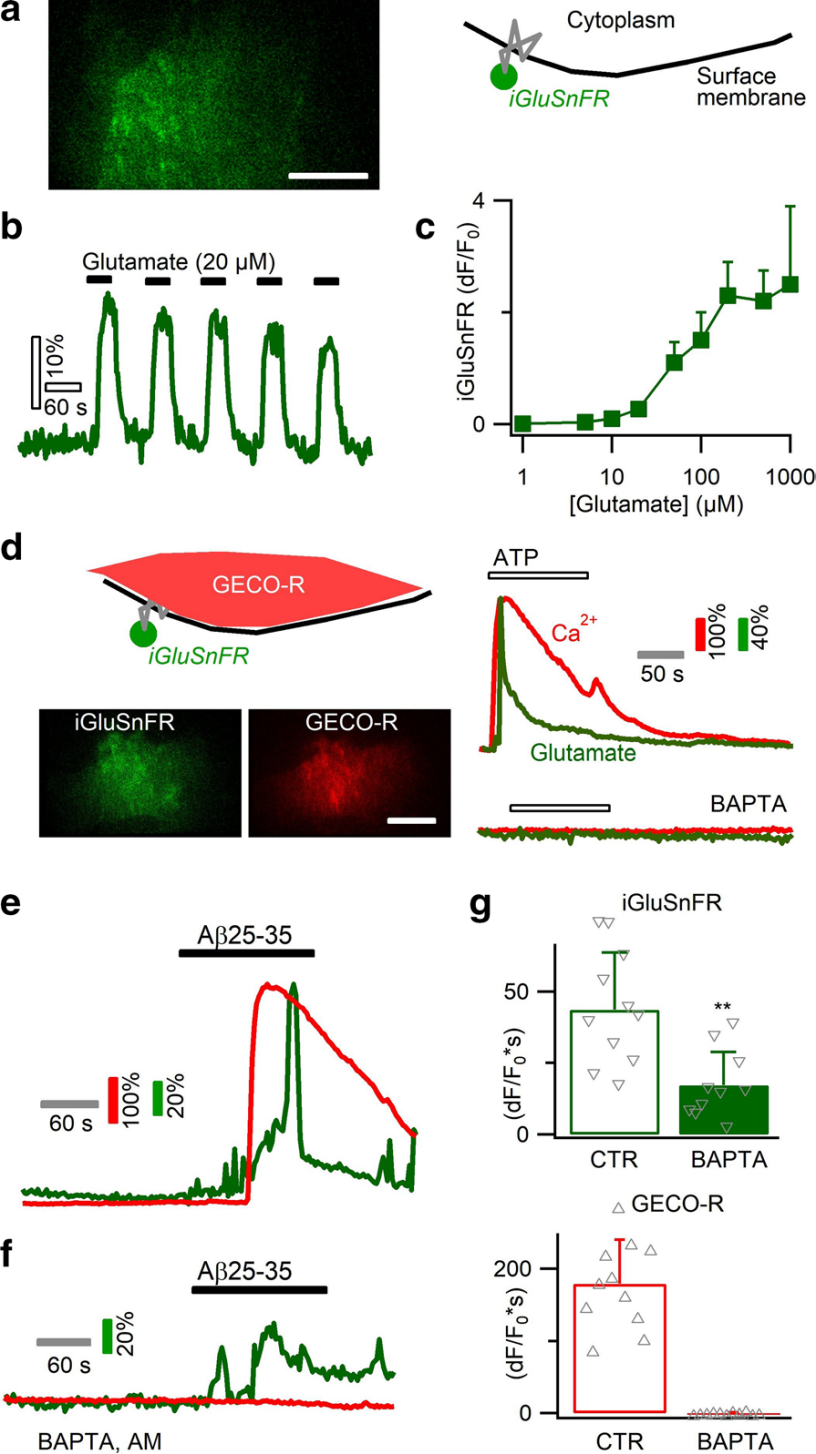
Biphasic astrocytic glutamate release occurring in both a Ca^2+^-dependent and -independent manner. **a** Imaging astrocytic glutamate release with green fluorescent sensor iGluSnFR expressed on the outer face of cell membrane. **b** CTR: repetitive fluorescence signals upon glutamate exposure. **c** Dose–response curve for astrocyte-expressed iGluSnR (n = 6–11 cells per concentration). **d** Dual-color imaging of astrocytic Ca^2+^ by the red sensor GECO-R and glutamate release by iGluSnFR. Right, ATP application evoked Ca^2+^ rise and glutamate release, which were both suppressed by BAPTA chelation of intracellular Ca^2+^. **e** Aβ25–35 triggered a biphasic glutamate release, which started prior to Ca^2+^ rise and was further increased during Ca^2+^ elevation. **f**, **g** Chelating astrocytic Ca^2+^ with BAPTA partially reduced Aβ-induced glutamate release, confirming its occurrence in both Ca^2+^-independent and -dependent manner (n = 10–11 cells per condition). Scale bars, 5 µm

Astrocyte Ca^2+^ signals have been suggested to trigger the release of signaling molecules and affect neuronal activity [5]. During pathological Aβ accumulation, neurotoxicity has been attributed to excessive buildup of extracellular glutamate [37, 54, 63]. To examine how astrocytes contribute to such glutamate buildup, we imaged astrocyte glutamate release in response to Aβ25–35. We expressed the genetically encoded glutamate sensor iGluSnFR in primary cortical astrocytes [51], which showed repetitive fluorescence change upon glutamate puff (Fig. 5a, b). The dose–response curve reveals a dynamic range of ~ 10–200 µM glutamate (Fig. 5c). Triggering astrocyte Ca^2+^ elevation by ATP caused glutamate release, which was inhibited by the Ca^2+^ chelator BAPTA AM (100 µM, Fig. 5d). We then observed that Aβ25–35 application also induced glutamate release as reflected by the green fluorescence increase of iGluSnFR (Fig. 5e). We noted that during Aβ application, a fraction of glutamate was released before the onset of Ca^2+^ elevation and the Ca^2+^ signal then accelerated the release (Fig. 5e). We then performed similar experiments in astrocytes loaded with the Ca^2+^ chelator BAPTA AM. While intracellular Ca^2+^ signal was fully inhibited, a portion of glutamate was still released upon Aβ25–35 application, thereby validating the presence of a Ca^2+^-independent release component (Fig. 5f, g). The presence of BAPTA, meanwhile, also reduced the total amount of glutamate release, showing the co-expression of Ca^2+^-dependent release (integral dF/F_0_*s = 17.2 ± 16.2 vs. CTR 43.6 ± 22.4; *p* < 0.01; Fig. 5g). Thus, Aβ25–35 induced astrocytic glutamate release via both Ca^2+^-dependent and -independent mechanisms.

### CX hemichannel affects Ca^2+^-independent glutamate release

Glutamate is known to permeate through hemichannels [64, 97]. We confirmed this by artificially opening CX hemichannels in cultured astrocytes with Ca^2+^-free solution [97], which indeed induced glutamate release (Additional file 1: Fig. S4). This occurred in the absence of intracellular Ca^2+^ increase, suggesting that CX hemichannels may contribute to the glutamate release preceding the Ca^2+^ rises.

**Fig. 6:**
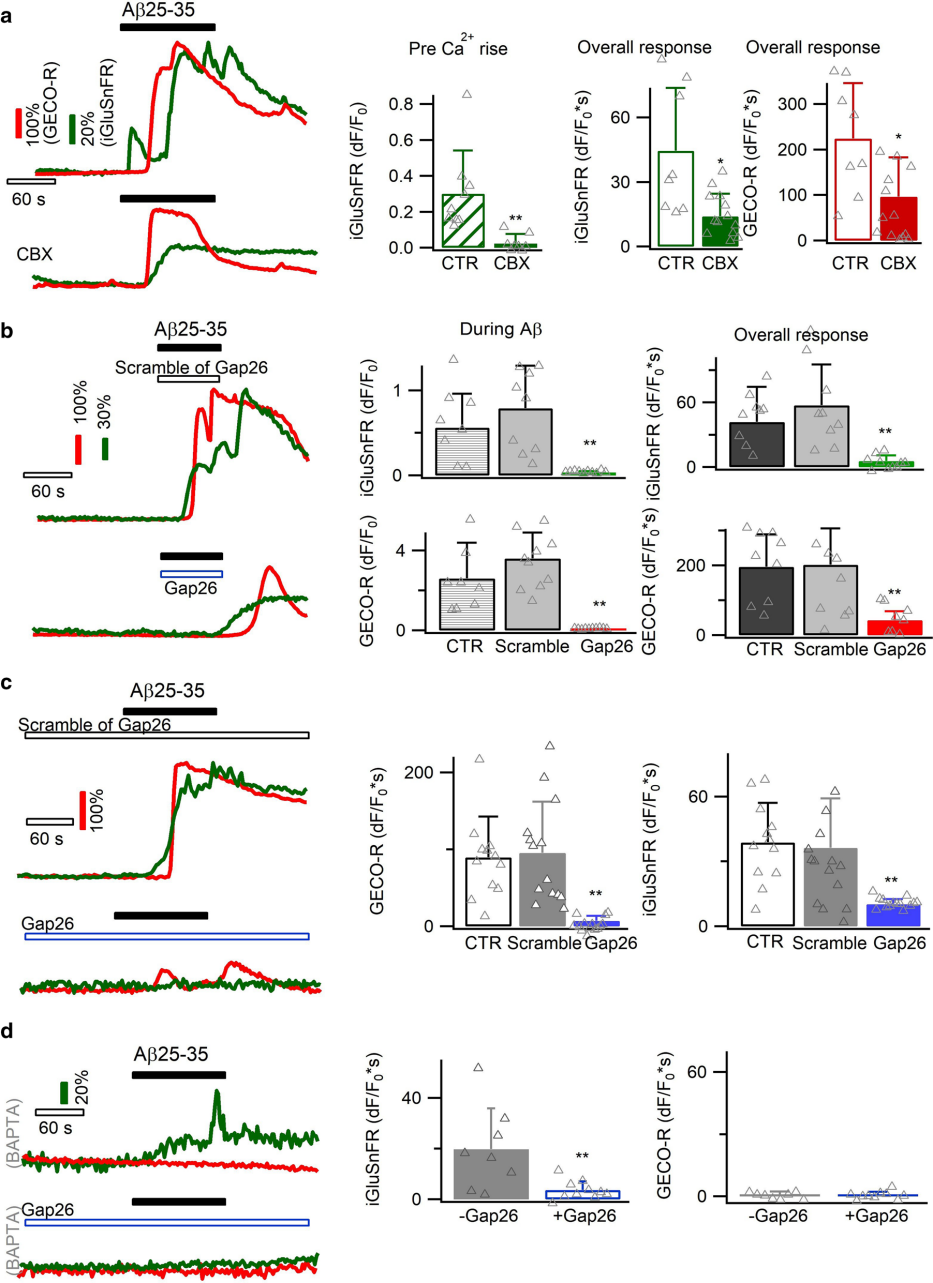
Involvement of CX hemichannels in Ca^2+^-independent glutamate release. **a** Glutamate release prior to the Ca^2+^ elevation (top, CTR) was reduced by the CX hemichannel blocker CBX (100 µM; present throughout the recording; n = 8–13 cells per condition). **b** During the Aβ application phase, Ca^2+^-independent glutamate release was blocked by another CX hemichannel blocker Gap26 peptide (200 µM). The inactive scramble peptide of Gap26 showed no effect (n = 8–10 cells per condition). **c** A more pronounced inhibition effect of Gap26 was observed when applying it throughout the entire imaging period (i.e., pre-, during- and post-Aβ; n = 12–14 per condition). **d** Chelating astrocytic Ca^2+^ with BAPTA AM isolated Ca^2+^-independent astrocytic glutamate release, which was inhibited by CX hemichannel blocker Gap26 (n = 8–10 per condition)

We next imaged Aβ-evoked glutamate release in the absence and presence of the CX hemichannel blocker CBX (100 µM). As expected, this treatment reduced the glutamate release during the phase prior to Ca^2+^ increase (dF/F_0_ = 0.03 ± 0.7 vs. 0.31 ± 0.24 of CTR; *p* < 0.01; Fig. 6a). The overall Ca^2+^ signal and glutamate release throughout the recording period were also reduced (Fig. 6a), corroborating that hemichannel opening contributes to the Aβ-evoked Ca^2+^ signal. Applying during Aβ25–35 stimulation the mimetic peptide Gap26 (200 µM), a selective blocker of connexin43 hemichannel [20, 64], also inhibited the Ca^2+^ elevation and glutamate release as compared to control and to the inactive scrambled peptide of Gap26 (Fig. 6b). Upon the washing of Gap26 and Aβ, glutamate release and Ca^2+^ signal reappeared (Fig. 6b), suggesting a post-inhibition rebound response. Applying Gap26 throughout recording (i.e., pre-, during- and post-Aβ application) inhibited the post-Aβ response (Fig. 6c). We then suppressed astrocyte Ca^2+^ signal with BAPTA and isolated the Aβ-induced Ca^2+^-independent glutamate release that was found to be affected by Gap26 (temporal integral dF/F_0_*s = 3.7 ± 3.4 vs. 19.7 ± 16.1 of CTR, *p* < 0.01; Fig. 6d). Hence, astrocyte CX hemichannel contributes to the Ca^2+^-independent glutamate release induced by Aβ25–35.

### Anion channel and lysosome exocytosis modulate Ca^2+^-dependent glutamate release

**Fig. 7:**
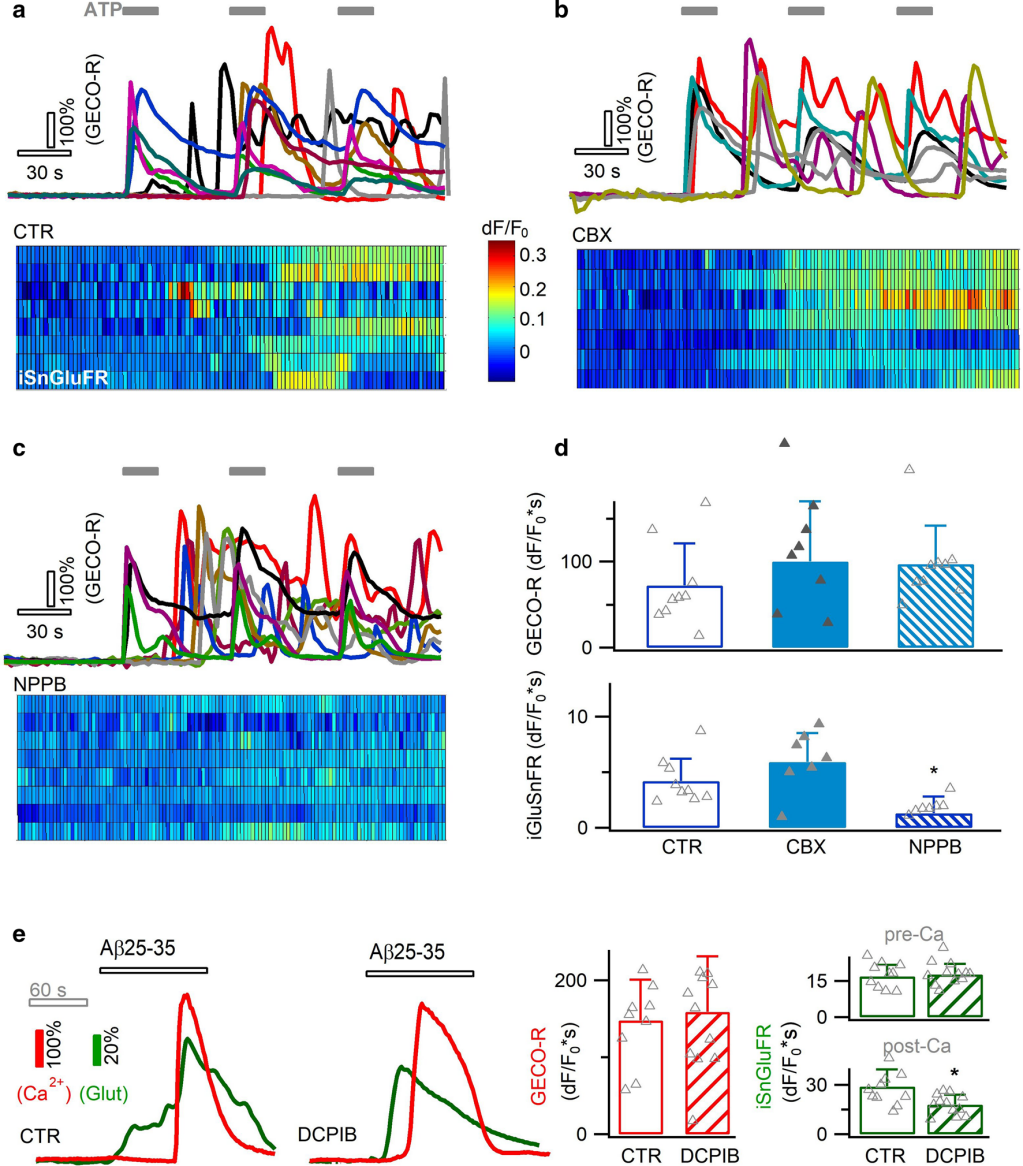
Contribution of anion channels to Ca^2+^-dependent glutamate release. **a** Aβ-evoked astrocytic Ca^2+^ rises were due to purinergic receptor activation. To examine Ca^2+^-dependent glutamtae release, we applied ATP (30 µM) to trigger astrocytic Ca^2+^ and glutamate release. **b**–**d** Ca^2+^-dependent glutamate release was unaffected by inhibition of CX hemichannel (CBX, 100 µM), but reduced by blocking anion channels with NPPB (100 µM) (n = 7–9 cells per condition). **e** During the response to Aβ25–35 (6 µM), inhibiting anion channels with DCPIB (50 µM) influenced the glutamate release during Ca^2+^ elevation phase (n = 10–12 cells per condition)

Astrocyte CX hemichannel opening was suggested to be Ca^2+^-sensitive [15], and may modulate Aβ-evoked Ca^2+^-dependent glutamate release. One way to clarify this issue would be to block CX hemichannels, which however is known to interfere with Aβ-induced Ca^2+^ signal (Figs. 2, 6). To bypass this problem, we mimicked Aβ-evoked Ca^2+^ signals by ATP stimulation (30 µM, Fig. 7a) that partially recapitulated the purinergic receptor activation by Aβ25–35 (Fig. 2). ATP-triggered Ca^2+^ oscillations showed comparable patterns as the Aβ25–35-indcued signals (GECO-R, Fig. 7a) and triggered glutamate release (iGluSnFR, Fig. 7a). Yet contrary to our expectation, Ca^2+^-activated glutamate release persisted in the presence of the CX hemichannel blocker CBX (100 µM, Fig. 7b, d), suggesting its dispensable involvement in this process. Alternatively, Ca^2+^-sensitive anion channels represent another route for Ca^2+^-activated glutamate release in astrocytes [29, 86, 93, 96]. Indeed, the anion channel blocker 5-nitro-2-(3-phenylpropylamino) benzoic acid (NPPB, 100 µM) reduced the Ca^2+^-activated glutamate release (temporal integral dF/F_0_*s = 1.3 ± 1.5 vs. 4.2 ± 1.9, *p* < 0.05; Fig. 7c, d). Also, during the response to Aβ25–35, astrocyte glutamate release over the phase of Ca^2+^ rise was reduced by another anion channel blocker DCPIB (50 µM, Fig. 7e), corroborating a role for anion channels in the Ca^2+^-dependent glutamate release.

**Fig. 8:**
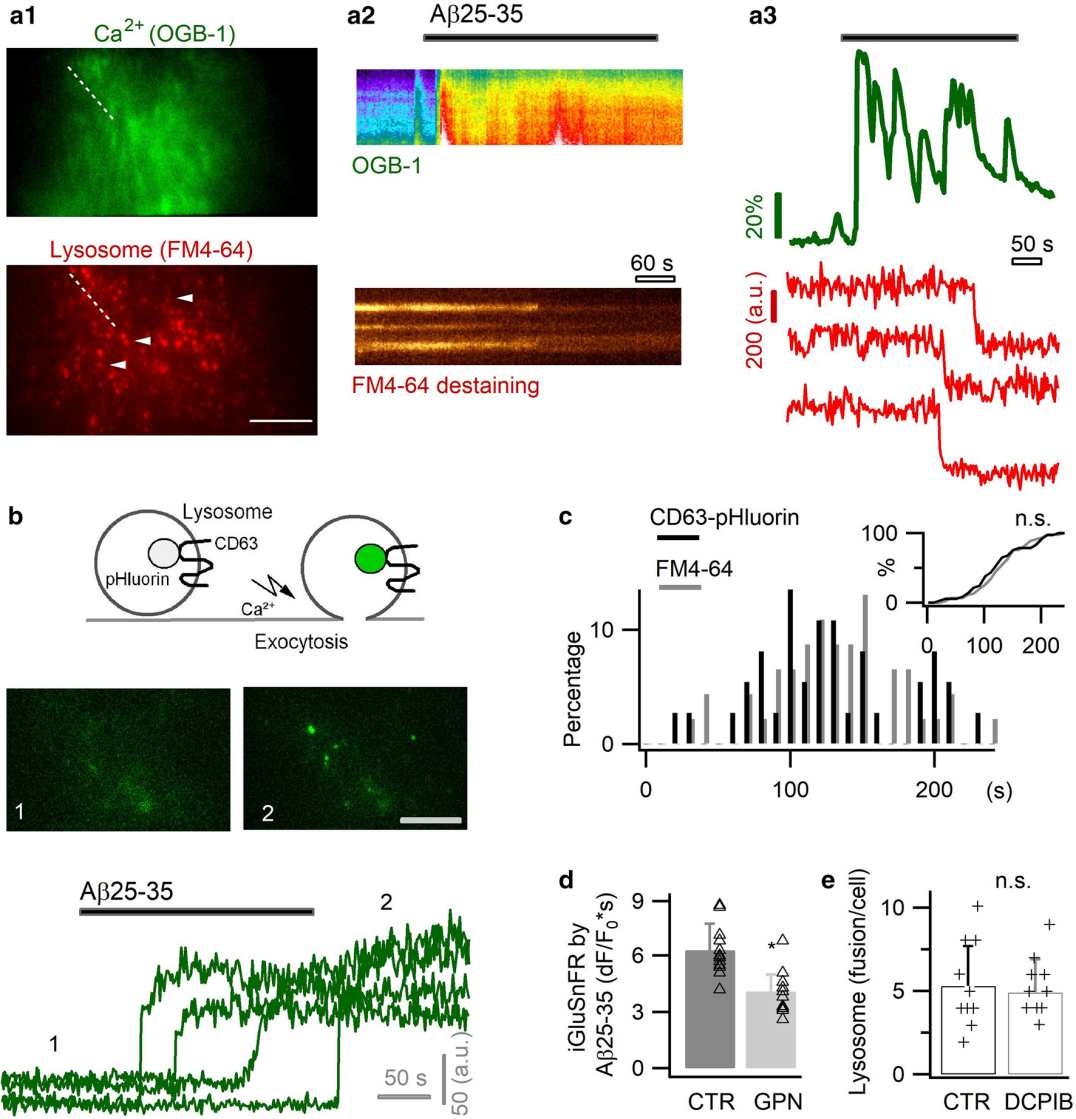
Aβ25–35 triggered astrocytic lysosome exocytosis. **a** Astrocytes co-labeled with the green fluorescent Ca^2+^ indicator OGB-1 AM and the red-fluorescent lysosomal marker FM4-64 (**a1**). Application of Aβ25–35 (6 µM) evoked Ca^2+^ elevation followed by asynchronous exocytosis of lysosomes, as reflected by FM dye destaining (**a2**, **a3**). **b** Aβ-evoked lysosomal exocytosis imaged with CD63-pHluorin. **c** Temporal distribution of lysosomal exocytosis obtained with FM dye and CD63-pHluorin (n = 51–62 lysosomes from five cells per condition). *Inset*, cumulative histogram showing the temporal coincidence for the two lysosomal markers (*p* = 0.7). **d** Permeabilization of lysosomes by GPN affected the Aβ25–35-induced glutamate release (iGluSnFR, dF/F_0_*s; n = 12 cells per condition; recording protocol is as Fig. 7e). **e** The presence of anion channel blocker DCPIB did not affect astrocyte lysosome release rate as measured by FM4-64 destaining (n = 10 cells per condition). Scale bars, 10 µm for **a**, 5 µm for **b**

Another pathway for glutamate release could be Ca^2+^-regulated vesicular exocytosis. In astrocytes, lysosomes are known vesicular compartments undergoing Ca^2+^-activated exocytosis [38, 50, 101], though the involvement of small synaptic like vesicles is still debated [23, 75]. The vesicular glutamate transporter sialin [58] was observed on astrocyte lysosomes [50], suggesting their contribution to Ca^2+^-dependent glutamate release. We therefore imaged with TIRFM lysosome exocytosis from primary astrocytes in response to Aβ25–35. Lysosomes were labeled with the red fluorescent dye FM4-64 [50, 101], and the concomitant Ca^2+^ signals monitored with the green fluorescent indicator OGB1 AM (Fig. 8a1). Following Aβ-evoked Ca^2+^ elevation, we observed an asynchronous lysosome exocytosis as reflected by FM dye destaining (Fig. 8a2, a3). We also used a pH-sensitive sensor to image exocytosis, where the GFP mutant pHluorin is conjugated to the intralumenal site of the lysosomal membrane protein CD63 [49] (Fig. 8b). CD63-pHluorin is quenched in the acidic lysosome lumen, and becomes fluorescent upon its exocytotic exposure to extracellular neutral solution. We observed that Aβ25–35 induced CD63-pHluorin brightening on astrocyte surface, thus corroborating the occurrence of lysosome exocytosis (Fig. 8b). A similar temporal distribution was found with the two exocytotic probes (*p* = 0.6; Fig. 8c), consistent with the co-localization of FM4-64 and CD63 in astrocyte lysosomes [50]. Next, to examine the potential glutamate storage in astrocyte lysosomes, we performed glutamate staining in cultured astrocytes that resulted in a punctuate labelling distributed across the cytoplasm (Additional file 1: Fig. S5a). Glutamate staining was diminished by the cathepsin C substrate glycyl-l-phenylalanine 2-naphthylamideto (GPN, 200 µM), a compound permeabilizing lysosomes by osmotic swelling [50, 101] (Additional file 1: Fig. S5a). On the other hand, the fluorescent nucleotide marker MANT-ATP showed little colocalization with FM4-64-labeled lysosomes (Additional file 1: Fig. S5b). It was also observed that permeabilization of lysosomes by GPN reduced the Aβ25–35-induced glutamate release (Fig. 8d), and the presence of anion channel blocker DCPIB (50 µM) showed no significant effect on astrocyte lysosome release (Fig. 8e). These data suggest the astrocyte lysosomes, in parallel with anion channels, contribute to Aβ-induced Ca^2+^-dependent glutamate release.

## Discussion

In this study, we examined the sub-cellular mechanisms underlying the astrocytic response to the neurotoxic amyloid beta fragment. A state-dependent alteration of Ca^2+^ homeostasis in association with a multiphasic release of signaling transmitters have been observed in primary cortical astrocytes. As illustrated in Additional file 1: Fig. S6a, the free cytosolic Ca^2+^ concentration is maintained at physiological low level (~ 100 nM) in astrocyes in basal conditions [62], thereby restraining the initial PMCA reaction to Aβ25–35 application. One possibility is that Aβ25–35 opens CX hemichannels leading to glutamate and ATP co-release, the latter triggering Ca^2+^ elevation to cause further glutamate release. In basal conditions, Aβ25–35 plays an excitatory role in upregulating astrocyte Ca^2+^ signals. In Aβ-preconditioned astrocytes (Additional file 1: Fig. S6b), the chronically overloaded intracellular Ca^2+^ sets a greater driving force for its efflux. Hence, PMCA Ca^2+^ export is readily activated by subsequent acute Aβ challenge, leading to an overshoot drop in the basal Ca^2+^ diminution and concomitant H^+^ influx. H^+^ then exerts an inhibitory effect on CX hemichannel opening, thereby blocking the hemichannel and the purinergic activaiton of Ca^2+^ elevation. In this situation, Aβ tends to exert an inhibitory effect on astrocyte Ca^2+^ signal. Immunostaining of PMCA and CX43, a major hemichannel protein expressed in astrocyes, was observed in cultured astrocytes and in the cortex of hAPPJ20 AD mouse model.

Dysregulation of neuron-glia interaction emerges as an important aspect in Aβ pathology and the evolution of AD [33]. Aberrant Ca^2+^ signals have been noted as a hallmark of astrocyte functional remodeling in AD mouse models [17, 47]. Our current data support the potential contribution of astrocytes to the dysregulated neuroglial activities in amyloidopathy, for instance via the interference with purinergic and/or glutamatergic communications [54]. The primary culture of astrocytes is an in vitro model to study the sub-cellular mechanism involved in AD pathophysiology. Primary astrocytes were reported to display a portion of properties different from their in vivo counterparts, like the genes featuring the reactive state [9, 100]. In the current study, preconditioning primary astrocytes with submicromolar Aβ25-35 caused appreciable alteration in their subsequent response to high-dose Aβ, indicating a malleable adaptability in their functional status. This suggests that the cultured astrocytes used in this study were not fully reactivated, likely mirroring an early state during brain Aβ deposition.

We show that Aβ25–35 activates Ca^2+^ elevation via purinergic P2Y1 receptor activation that confirms the in vivo finding in AD mouse model [17]. The involvement of the Ca^2+^ release from the internal ER store is also in line with the previous in vitro study [85]. ER Ca^2+^ depletion is followed by Ca^2+^ influx via SOC channel [66], which was here observed to be facilitated by Aβ25-35, as previously reported with Aβ42 [74]. This therefore provides an additional mechanism for the upregulated astrocyte Ca^2+^ signal and explains in part its dependence on Ca^2+^ influx. We also show that Aβ25–35-caused Ca^2+^ signals depend on the opening of CX hemichannels, a major pathway for ATP release from astrocytes [28, 98]. It is possible that Aβ25–35 triggers ATP release from CX hemichannels that then activates astrocyte P2Y1 receptor to cause Ca^2+^ elevation. Interestingly, purinergic autocrine stimulation and subsequent glutamate release has also been observed following optogenetic activation of astrocytes with channelrhodopsin 2 [79]. Optical activation of astrocytes with light-gated GPCRs optoAR and melanopsin also triggered ATP release and autocrine activation of astrocytic purinergic receptors [25, 53]. Nevertheless, our current data could not fully exclude other possible mechanisms underlying Aβ25–35-induced Ca^2+^ signals. For instance, Aβ25–35 may directly activate astrocyte purinergic receptors, which could be mitigated by CX hemichannel blocking.

Besides the generally observed excitatory effect on astrocytic Ca^2+^ of Aβ peptides or plaques, we observed an inhibitory effect of Aβ25–35 in astrocytes preconditioned by submicromolar concentrations of Aβ. It was reported that Aβ25–35 not only triggered Ca^2+^ elevation, but also inhibited ATP-evoked Ca^2+^ elevation in primary cultures of rat astrocytes [85], implying a mixed status of the astrocytes used therein. We here attributed the mechanism of inhibition to the potentiation of PMCA-mediated Ca^2+^ extrusion from the cytoplasm, modulated by Aβ-triggered cAMP elevation. As ATP-driven pumps, PMCAs export cytosolic Ca^2+^ in a calmodulin-dependent manner to maintain its physiological low level [8, 62]. Overexpression of a human PMCA in striatal astrocytes was used to inhibit Ca^2+^ signals [99]. In our study, the ready activation of PMCA by Aβ in preconditioned astrocytes implies that their cytosolic Ca^2+^ concentration, due to the gradual overload upon the chronic Aβ exposure, has been hyper-shifted from the physiological level. As H^+^ is a hemichannel inhibitor [77], the H^+^ influx that was coupled with PMCA-mediated Ca^2+^ extrusion would have inhibited CX hemichannel, an effect that we found to attenuate Ca^2+^ elevations. This thus resulted in a dominant inhibitory effect in preconditioned astrocytes. Ca^2+^ export by PMCA likely represents a protective mechanism to counterbalance the early Ca^2+^ upregulation in astrocytes caused by Aβ. Nevertheless, PMCA activity is often impaired by recurrent activation and metabolic stresses [8], as it would be encountered as a consequence of long-term Aβ accumulation in AD [48]. Hence, Ca^2+^ hyperactivity could become eventually prevalent in astrocytes at the time when Aβ plaques are formed [17, 47].

In an AD mouse model, astrocytes Ca^2+^ hyperactivity occurs globally independent of their proximity to Aβ plaques, suggesting that the local Aβ pathology is transmitted by intercellular mechanisms [47]. We here observed Aβ25-35-caused multiphasic release of glutamate from astrocytes, which could activate adjacent astrocytes and neurons in situ. Our data also suggest that Aβ25-35 likely causes ATP release via CX hemichannels to activate astrocyte Ca^2+^ elevation (Additional file 1: Fig. S6a). CX hemichannels are known to release signaling molecules from astrocytes regulating neural activity in physiological and pathological conditions [28]. In AD mouse models, CX hemichannels have been implicated in the release of ATP and glutamate, which dysregulate synaptic transmission [98]. We here suggest the mechanistic steps underlying Aβ-induced glutamate and ATP release. CX hemichannels may initiate the ATP release that subsequently activated purinergic autoreceptor to elevate Ca^2+^ signal. Consistent with CX hemichannels being nonselective channel pores [28], they were here also observed to mediate Ca^2+^-independent glutamate efflux. In addition, we noted that Aβ-evoked Ca^2+^ signal further increased glutamate release. While Ca^2+^-dependent glutamate release from astrocytes is being debated under physiological conditions [24, 78], it has been observed upon the hyper-regulated astrocyte Ca^2+^ signals in pathological conditions [89].

Our results suggest that Aβ-evoked Ca^2+^-dependent glutamate release occurs via astrocytic anion channels and lysosome exocytosis. Astrocyte glutamate release was suggested to be mediated by mouse Bestrophin 1 channel in a Ca^2+^-activated manner [67] (but see [94]). In APP/PS1 AD mouse model, excessive GABA release from astrocytic Bestrophin 1 channel was also observed to impair memory and learning [39]. In addition, SWELL-1 (i.e., LRRC8A) channel has been shown to constitute the anion channels that mediate glutamate release from astrocytes in association with cell swelling [96]. The relative roles of Bestrophin 1 and SWELL-1 in Aβ-evoked glutamate release needs be further evaluated. We also observed asynchronous lysosome exocytosis following Aβ-triggered Ca^2+^ signal, likely contributing to signaling molecule release. Lysosomes represent a population of vesicular compartments having a larger size than small secretory vesicles [49]. Although the physiological role of small secretory vesicles in astrocytes remains debated [75], lysosome exocytosis has been observed in response to pathological stimulations [21, 50, 84]. It is therefore plausible that lysosome exocytosis plays a role in modulating astrocytic signals in Aβ pathologies. Astrocytes could bidirectionally control synaptic transmission, for example via glutamatergic potentiation and purinergic/adenosinergic inhibition [12]. Hence, Aβ-evoked ATP and glutamate release would affect neuronal activities in situ during AD progression, with specific outputs depending on the receptor expression profiles of the local circuitry and their spatial correlation with astrocytic release sites. Aβ-induced glutamate release likely contributes to the glutamate neurotoxicity seen in AD context [90, 98]. Memantine, the uncompetitive antagonist with moderate affinity for the glutamate *N*-methyl-d-aspartate (NMDA) receptor, has been approved for the treatment of moderate to severe AD [68].

It has been noted that globally ablating pathologically altered astrocytes in AD mouse model worsens the disease [42]. Thus, understanding and hence being able to target dysregulations in specific signaling pathways in astrocytes holds the potential to ameliorate Aβ pathology. In this context, the current results provide testable targets to control astrocyte responses to neurotoxic Aβ peptide and will help to understand the astrocytic contributions.

## Supplementary Information

**Additional file 1. **Figure S1–S6 and Table S1–S2.

## Data Availability

The datasets used and analyzed during the current study are available from the corresponding author upon request.
