# Supplementary material for: Astrocytes respond to a neurotoxic Aβ fragment with state-dependent Ca2+ alteration and multiphasic transmitter release
Source: Acta Neuropathol Commun. 2021 Mar 16;9:44. doi: 10.1186/s40478-021-01146-1 (PMC7968286; doi:10.1186/s40478-021-01146-1)
Supplement: Supplementary file 1 — Additional file 1. Figure S1–S6 and Table S1–S2. [file 40478_2021_1146_MOESM1_ESM.pdf]

Supplementary material

Astrocytes respond to a neurotoxic Aβ fragment with state-dependent Ca^{2+} alteration and multiphasic transmitter release

Abbreviation list:

2-APB – 2-Aminoethoxydiphenyl borate
Aβ – amyloid β
AD – Alzheimer's disease
cAMP – cyclic adenosine monophosphate
CBX - carbenoxolone
CX – connexin
DCPIB – 4-[(2-butyl-6,7-dichloro-2-cyclopentyl-2,3-dihydro-1-oxo-1*H*-inden-5-yl)oxy]butanoic acid
EPAC – exchange protein directly activated by cAMP
ER – endoplasmic reticulum
FRET – fluorescence resonance energy transfer
GLT-1 – glutamate transporter 1
GLAST – glutamate aspartate transporter
GPN – glycyl-L-phenylalanine 2-naphthylamide
mGluR – metabotropic glutamate receptor
MPEP – 2-Methyl-6-(phenylethynyl)pyridine
NCX – sodium calcium exchanger
NPPB – 5-nitro-2-(3-phenylpropylamino) benzoic acid
OGB-1 – Oregon green bapta 1
PMCA – plasma membrane Ca^{2+} ATPase
PPADS – pyridoxalphosphate-6-azophenyl-2',4'-disulfonic acid
SOC – store-operated channel
SD – standard deviation
TIRFM – total internal reflection fluorescence microscopy

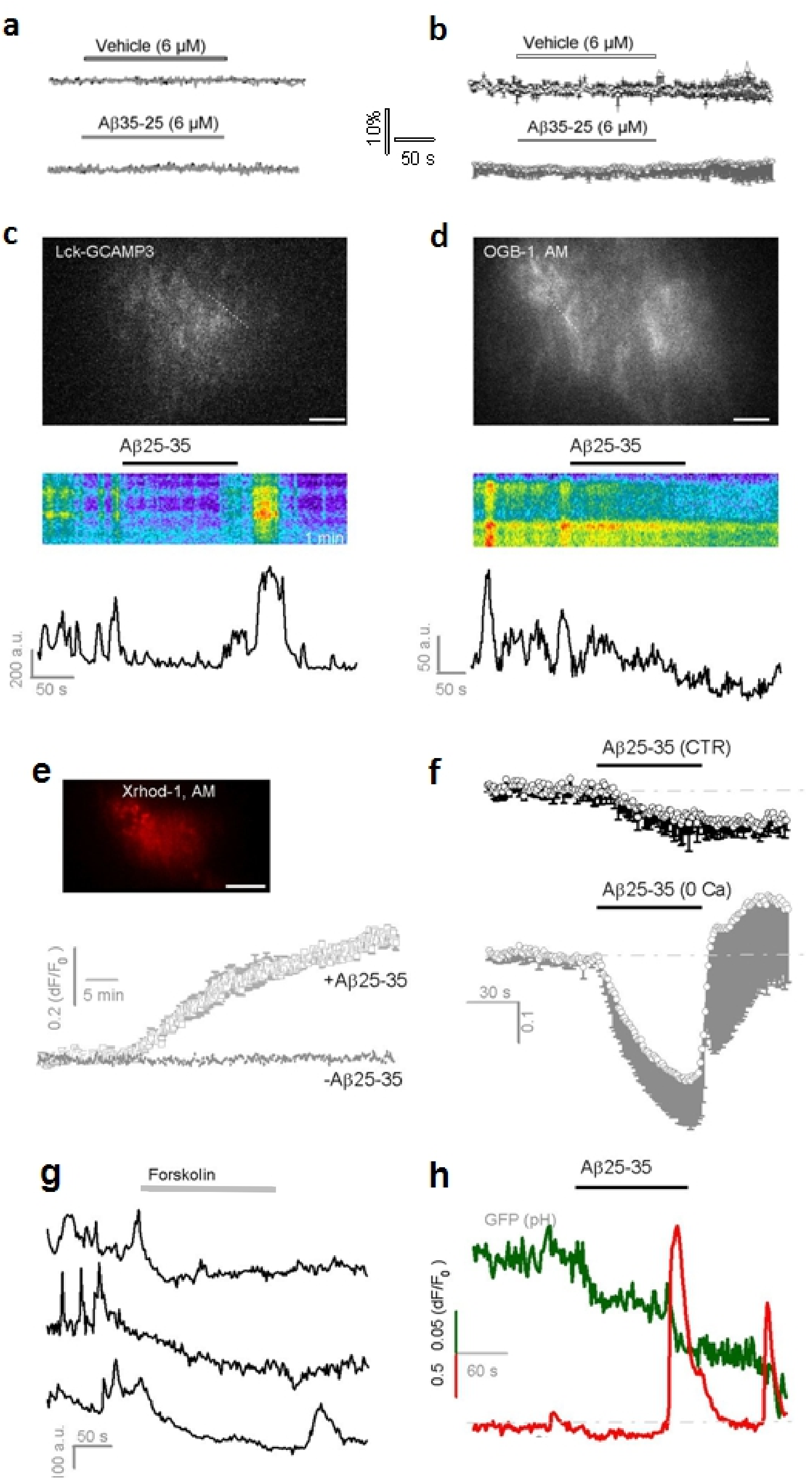

Fig. S1 Effects of Aβ25-35 on astrocyte Ca^{2+} signaling. Cultured astrocytes in basal condition (**a**) and after Aβ preconditioning (**b;** Aβ25-35, 0.5 μM, 2 hr) in response to either vehicle (i.e., equal molar H_2O) or the reversed peptide Aβ35-25 (n = 7 per condition). (**c-d**) Inhibitory effect of Aβ (6 μM) on spontaneous astrocytic Ca^{2+} oscillation. (**e**) Preconditioning with Aβ25-35 (0.5 μM) caused gradual Ca^{2+} overload in astrocytes. The chemical Ca^{2+} dye

Xrhod-1, AM was used for long-term Ca^{2+} imaging (n = 8 cells for each). (**f**) Removing extracellular Ca^{2+} facilitated Aβ25-35-caused Ca^{2+} diminution (n = 9 for CTR, n = 7 for zero external Ca^{2+} condition). (**g**) Inhibition of spontaneous astrocyte Ca^{2+} oscillations by forskolin (100 μM). (**h**) Stepwise decreases in intracellular pH observed in the 'mix' type astrocyte Ca^{2+} response, which was evoked by Aβ25-35 (6 μM) in astrocytes after short-term preconditioning (Aβ25-35 0.5 μM, 0.5 hr). The red Ca^{2+} sensor GECO-R and the pH-sensitive GFP fluorescent protein were co-expressed in cultured astrocytes. During Aβ25-35 application, the initial small diminution in basal Ca^{2+} was likely due to the direct potentiation of PMCA that meanwhile caused H^+ influx thereby decreasing the GFP fluorescence. The delayed Ca^{2+} rise would have also activated PMCA to confine the Ca^{2+} elevation, inducing more H^+ influx to further quench the GFP fluorescence. During the elevation phase, cytosolic Ca^{2+} could also be buffered into ER via the sacro/endoplasmic reticulum Ca^{2+} ATPase. Scale bars, 5 μm.

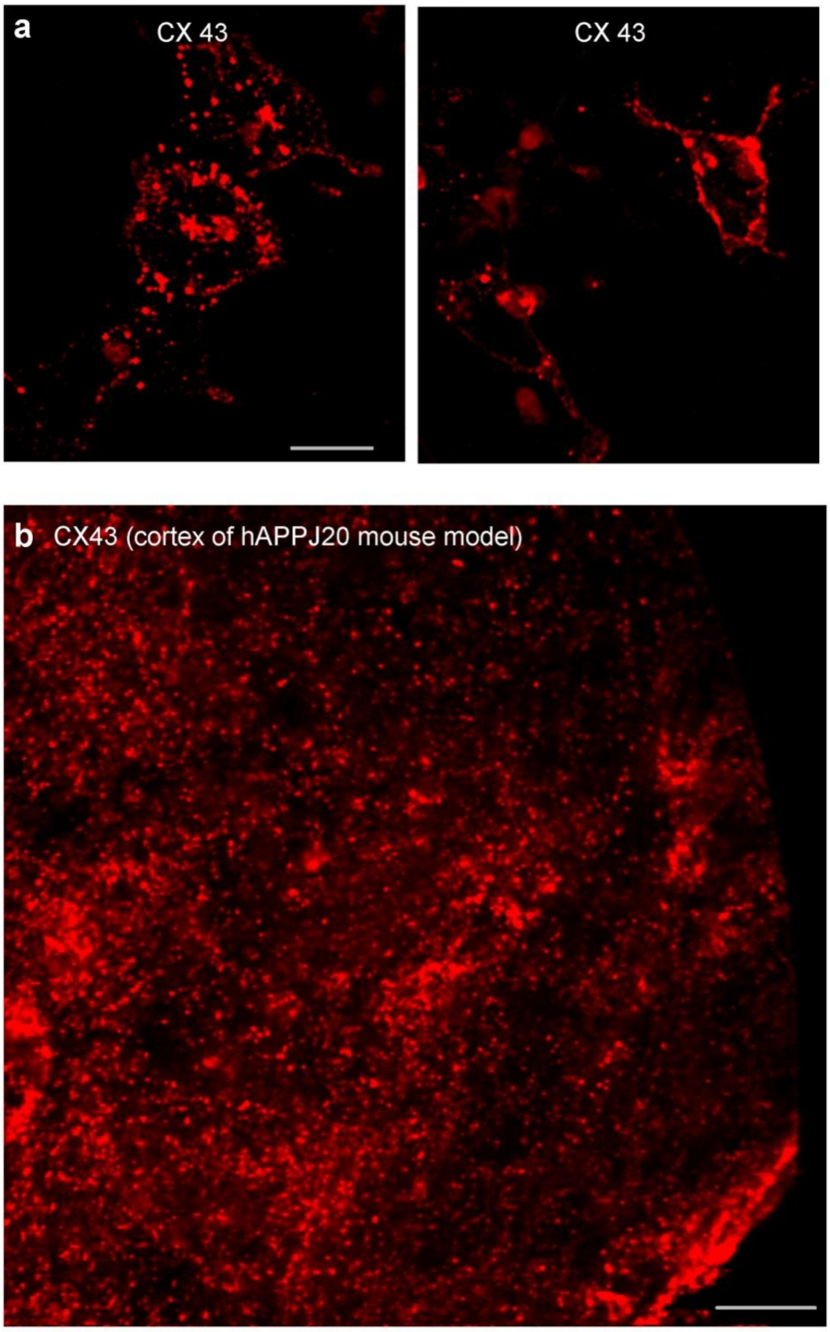

Fig. S2 Immunostaining of connexin 43 in cultured cortical astrocytes (**a**) and in somatosensory cortex of hAPPJ20 AD mouse (~7 month age; **b**). Scale bars, 20 μm.

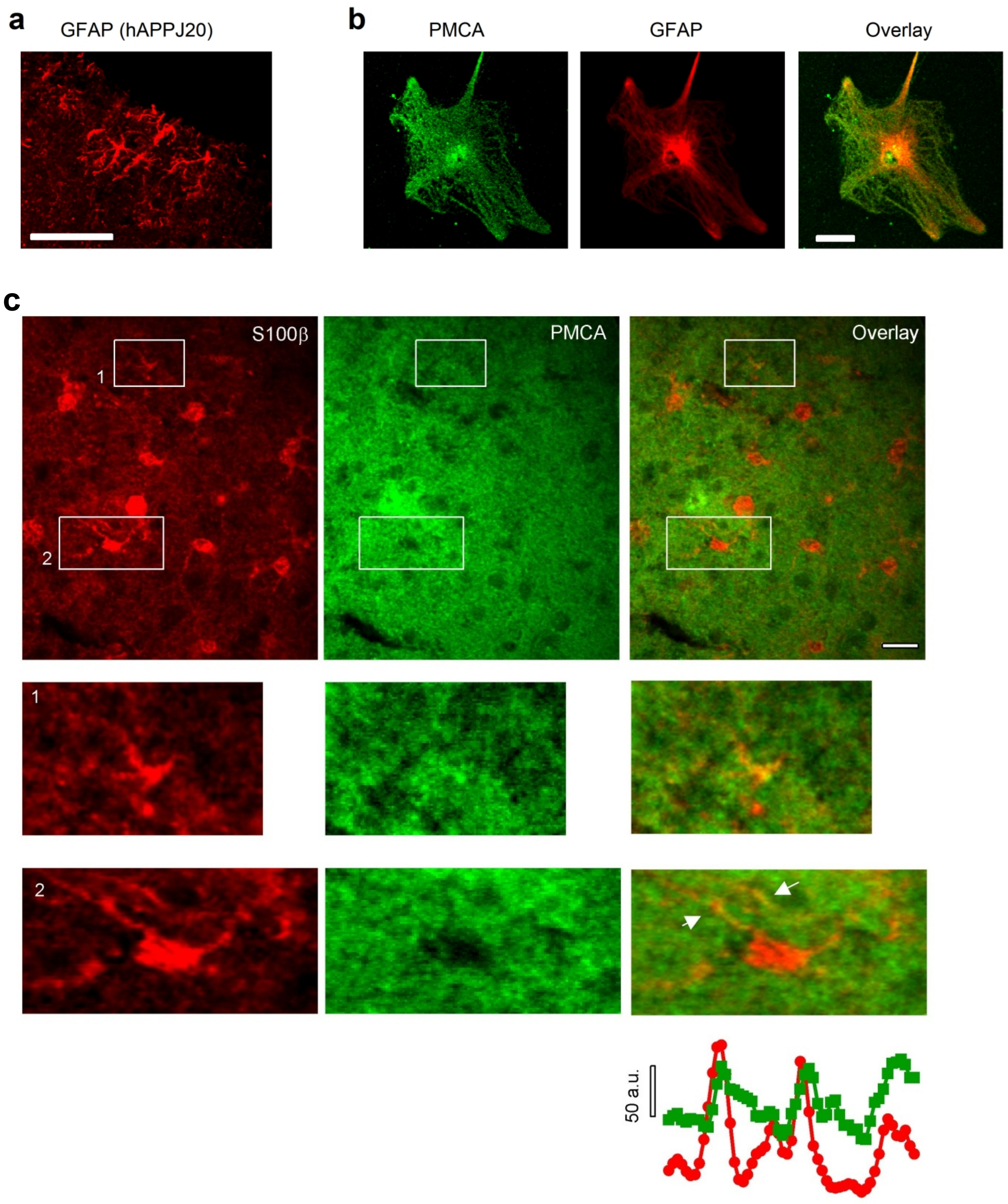

Fig. S3 Immunostaining of PMCA in astrocytes. (**a**) GFAP-positive astrocytes observed by immunostaining in the somatosensory cortex of J20 AD mouse model (~7 month old). Scale bar, 50 µm. (**b**) Immunostaining of a pan PMCA antibody against the astrocyte marker GFAP in cultured cortical astrocytes of wild-type mice. Scale bar, 10 µm. (**c**) PMCA

immunostaining in astrocytes in somatosensory cortex of hAPPJ20 AD mouse model. The diffuse staining pattern is consistent with the membranous expression of PMCA. A subpopulation of PMCA labeling was observed to surround S100β-positive astrocytic somata, and present in S100β-identified processes. Mouse age is ~7 month. Scale bar, 20 µm.

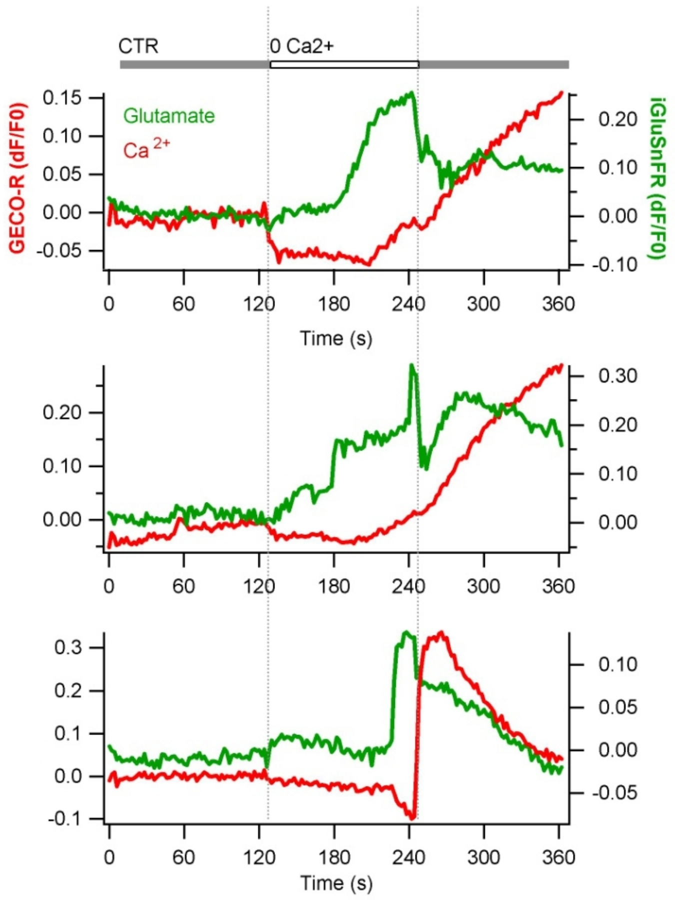

Fig. S4 Astrocyte glutamate release via opened CX hemichannels. CX hemichannels were opened by Ca^{2+}-free solution (open bar). Cytosolic Ca^{2+} and glutamate release were simultaneously imaged with TRIFM, in astrocytes co-expressing the red GECO-R and green iGluSnFR sensor. Glutamate release was observed in the absence of Ca^{2+} elevation. A diminution in cytosolic Ca^{2+} was observed in the phase of 0 Ca^{2+}, indicating the gradual Ca^{2+} efflux due to the reversed ion gradient. Switching back to control solution (1.8 mM Ca^{2+}) re-closed CX hemichannels, causing a transient inhibition for the glutamate release; meanwhile, re-supplying Ca^{2+} elevated Ca^{2+} levels in the cytosol that then sustained another

phase of glutamate release. This result is in line with the co-existence of Ca^{2+}-independent and -dependent glutamate release from astrocytes.

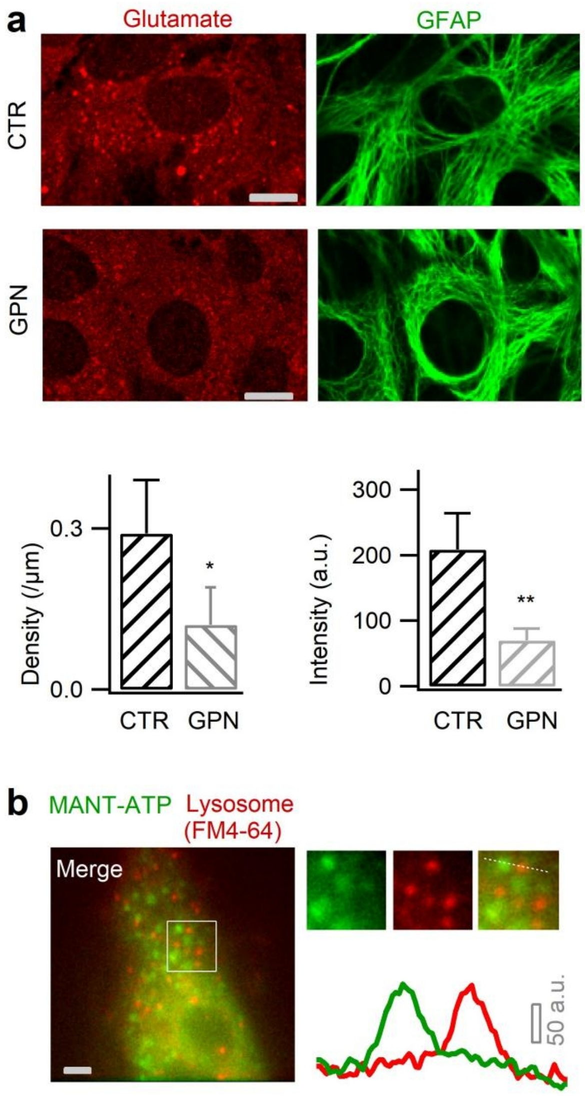

Fig. S5 Astrocyte lysosomes versus glutamate staining and ATP indicator. (**a**) Glutamate immunostaining in cultured astrocytes concentrated in perinuclear vesicular compartments. Punctate staining was reduced by cathepsin C substrate GPN (200 µM), a lysosome-disrupting compound (n = 9 - 11 cells per condition). Scale bar, 5 µm. (**b**) Representative spatial distribution of the fluorescent ATP marker MANT-ATP (50 µM, 1 hr) and the lysosomal marker FM4-64 (6.7 µM, 30 min). Scale bar, 10 µm.

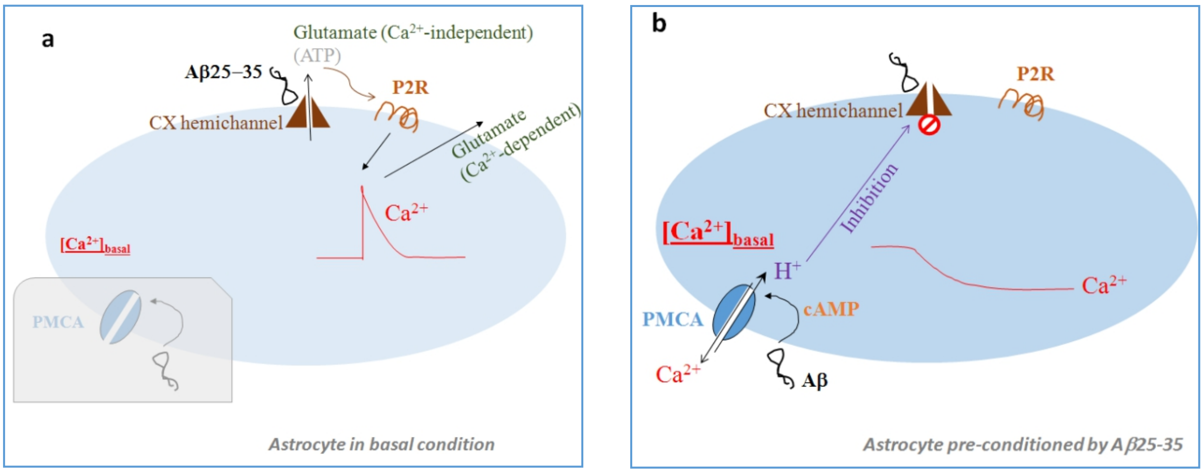

Fig. S6 Astrocyte response to Aβ25-35. (**a**) In astrocyes of basal conditions, cytosolic Ca^{2+} is maintainted at physiological low level (~100 nM) and PMCA shows no strong reaction to Aβ25-35. Meanwhile, Aβ25-35 acts on CX hemichannels leading to the release of glutamate, and likely ATP, in a Ca^{2+}-independent manner. Activation of purinergic P2 receptors then contributes to the intracellular Ca^{2+} elevation that triggers the Ca^{2+}-dependent release of glutamate. Aβ25-35 therefore plays an excitatory role in astrocytes in basal conditions. (**b**) Preconditioning of astrocytes with submicromolar Aβ25-35 peptide leads to gradual intracellular Ca^{2+} overload thereby setting a greater driving tendency for Ca^{2+} efflux. In this situation, PMCA actively reacts to subsequent Aβ25-35 stimulation, extruding cytosolic Ca^{2+} to the extracellular space therefore lowering the intracellular Ca^{2+} level. The coupled H^+ influx then inhibits the opening of CX hemichannels and the intracellular Ca^{2+} elevation. Aβ25-35 hence exerts an inhibitory effect on the inracellular Ca^{2+} level in preconditioned astrocytes.

Table S1 Antibodies used for fluorescence immunostaining

Experiments	Primary antibodies	Secondary antibodies
**Single staining**		
GFAP in brain slices of hAPPJ20 AD mouse model (Figure 3i)	Rb GFAP, Agilent/DAKO, Cat N° Z0334, 1/1000	Alexa 594 Goat (G) anti-Rb, Invitrogen, Cat N° A11012, 1/1000
Connexin 43 staining in brain slices of hAPPJ20 AD mouse model and in cultured astrocytes (Figure S2)	Rb Connexin 43, Sigma, Cat N° C6219, 1/1500	Alexa 594 G anti-Rb, Invitrogen, Cat N° A11012, 1/1000
**Double staining**		
GFAP and PMCA in cultured astrocytes (Figure 3j)	Rb GFAP, Agilent/DAKO, Cat N° Z0334, 1/1000<br><br>Mouse (M) pan PMCA ATPase Monoclonal Antibody, ThermoFisher Scientific, Cat N° MA3-914, 1/500	Alexa 594 Goat (G) anti-Rb, Invitrogen, Cat N° A11012, 1/1000<br><br>Alexa 488 G anti M IgG2A, Invitrogen, Cat N° A-21131, 1/1000
S100β and PMCA co-staining in the cortex of J20 AD mouse (Figure S3)	Rb monoclonal S100β , Abcam, Cat N° ab52642, 1/500<br><br>Mouse (M) pan PMCA ATPase Monoclonal Antibody, ThermoFisher Scientific, Cat N° MA3-914, 1/500	Alexa 594 G anti-Rb, Invitrogen, Cat N° A11012, 1/1000<br><br>Alexa 488 G anti M IgG2A, Invitrogen, cat N°A-21131, 1/1000

Table S2 Combinations of excitation wavelengths and filters for TIRFM imaging

**Fluorophore**	Excitation laser line (nm)	Dichroic filter (nm)	Emission filter (nm)
EGFP, pHluorin	488	500LP or PolyX[§]	535(50)BP
GECO-R	568	PolyX[§]	600LP
GFP(nd)-EPAC1(dDEP)-mCherry	488	PolyX[§]	535(50)&600LP[$]
OGB-1	488	PolyX[§]	535(50)BP
OGB-1 and FM4-64	488	PolyX[§]	535(50)&675(50)BP[$]
Xrhod-1	568	PolyX[§]	600LP or 615(45)BP

[§]PolyX – custom dual dichroic mirror with 488/568/NIR reflection bands and low ripple high-transmission elsewhere (AHF Analysentechnik).
[$]*denotes filters for simultaneous view with a custom dual-view device*.
